# ULK1 knockout suppresses pancreatic cancer progression by inhibiting autophagy and enhancing antitumor immunity

**DOI:** 10.1038/s12276-025-01590-2

**Published:** 2025-12-17

**Authors:** Hana Jeong, Jinju Lee, Ji-Yoon Son, JinKyung Lee, Miju Kang, Sunghyeon Cho, Ji Hyeon Kim, Yoon Jeon, Jonghyun Lee, Dongkwan Shin, Hye-Ran Kim, Ho Lee, Heesun Cheong

**Affiliations:** 1 Cancer Metastasis Branch, Division of Cancer Biology, Research Institute National Cancer Center, Goyang, Republic of Korea; 2 Department of Cancer Biomedical Science, National Cancer Center Graduate School of Cancer Science and Policy, Goyang, Republic of Korea; 3 Bioinformatics Branch, Division of Cancer Data Science, Research Institute, National Cancer Center, Goyang, Republic of Korea

**Keywords:** Cancer models, Macroautophagy, Tumour immunology

## Abstract

Autophagy plays a dual role in cancer, acting as a tumor suppressor and promoter depending on tumor stage and context. While Atg5 and Atg7 are well established core autophagy genes, the role of Unc-51-like kinase 1 (ULK1)—a key autophagy initiator—remains poorly understood in pancreatic ductal adenocarcinoma (PDAC). Here we investigated the role of ULK1 using tissue-specific deletion in genetically engineered mouse models. Although ULK1 messenger RNA levels remained unchanged between normal and tumor cells in The Cancer Genome Atlas dataset, multiplex immunohistochemistry revealed elevated ULK1 activity, marked by pATG14, in high-grade human PDAC tissues. Genetic deletion of Ulk1 impaired autophagy and reduced cell proliferation, colony formation and invasiveness of pancreatic cancer cells. In vivo, both syngeneic orthotopic and KPC (*LSL-Kras*^*G12D*/+^*; LSL-Trp53*^*R172H*/+^*; Pdx1-Cre*) mouse models with tissue-specific Ulk1 deletion exhibited significant delayed tumor progression, reduced tumor burden and extended survival. Importantly, Ulk1 deficiency remodeled the tumor immune microenvironment by reducing tumor-promoting polymorphonuclear myeloid-derived suppressor cells and neutrophils while substantially enhancing recruitment of cytotoxic CD8^+^ T cells and major histocompatibility complex II^+^ antigen-presenting cells. Chemokine and cytokine profiling revealed that downregulation of Cxcl2, Ccl2 and G-CSF might lead to polymorphonuclear myeloid-derived suppressor cell and neutrophil recruitment and survival, with concurrent upregulation of GM-CSF for dendritic cell infiltration, thereby inducing antitumor immunity. These findings provide insights into the role of ULK1 in PDAC progression through tumor-intrinsic metabolic support by autophagy activation and immune modulation by tumor-derived cytokines. Targeting ULK1 may represent a promising therapeutic strategy by inhibiting autophagy and enhancing antitumor immune responses in pancreatic cancer.

## Autophagy initiator ULK1 promotes tumor growth in pancreatic ductal adenocarcinoma

This study explores the role of a protein called ULK1 in pancreatic cancer. ULK1 is involved in autophagy. Researchers wanted to understand how ULK1 affects cancer progression, especially in pancreatic ductal adenocarcinoma. The team used mouse models and cell experiments to study the function of ULK1. They created mice with a specific deletion of the ULK1 gene in their pancreas and observed the effects on tumor growth. They also used CRISPR–Cas9 technology to knock out ULK1 in cancer cells. Their findings showed that removing ULK1 slowed down tumor growth and altered the immune environment around the tumor. This change led to fewer immune-suppressing cells and more immune-activating cells. The study concludes that targeting ULK1 could be a promising strategy for treating pancreatic cancer by both inhibiting autophagy and enhancing the body’s immune response against tumors.

This summary was initially drafted using artificial intelligence, then revised and fact-checked by the author.

## Introduction

Autophagy is a conserved intracellular degradation pathway that sustains cellular homeostasis by recycling macromolecules and clearing damaged organelles. Autophagy plays a crucial role in balancing normal cell functions and multiple pathophysiological conditions, including cancer. While it preserves genome stability in early cancer, it later supports tumor survival by maintaining organelles, enabling recycling and fulfilling metabolic demands^1,2^.

The tumor-promoting role of autophagy has been validated in genetically engineered mouse (GEM) models. Oncogenic mutations in *HRas* or *KRas* increase basal autophagy, which is necessary for tumor cell survival during tumorigenesis^3^. Deletion of *Atg5* or *Atg7* in KRas^*G12D*^-driven spontaneous lung cancer models reduced tumor size compared with that in wild type (WT) mice^4,5^. Melanomas with BRAF mutations^6^ and pancreatic cancers harboring 95% *KRas* mutations also exhibit high dependency on autophagy genes such as *Atg5* and *Atg7*^7,8^. In addition, depleting Atg proteins such as FIP200, Atg16l or Atg4 suppresses tumor growth in various cancers^9–11^. Accordingly, autophagy inhibition has been considered a promising therapeutic strategy^7,8^.

Unc-51-like kinase 1 (ULK1), a serine–threonine kinase, has gained attention as a druggable target, which plays a crucial role in the initiation step of autophagy^12^. ULK1 forms a complex with proteins such as ATG13, ATG101 and FIP200/RB1CC1, facilitated by the phosphorylation of ATG13^13–15^, and subsequently phosphorylates the components of the class III phosphoinositide 3)^3^ Kinase (PIK3C3)/Vps34 complex, including ATG14 and Beclin1 to drive autophagosome formation^16–18^. ULK1 activity is tightly controlled by upstream nutrient-signaling molecules such as mTORC1 and AMPK, which phosphorylate ULK1 at distinct residues to either inhibit or enhance its function^19^. The ULK family includes ULK1, ULK2, ULK3, ULK4 and STK368, with ULK1 and ULK2 showing the highest similarity^20^. However, ULK1 is the dominant initiator of autophagy, as seen in *Ulk1*^−/−^ *Ulk2*^−/−^ double-knockout (KO) mice, which exhibit severe neonatal mortality and autophagy defects similar to other core *Atg* gene deletions^21–24^. Although the cellular functions of ULK1 have been studied in several diseases, direct evidence of the role of ULK1 on the tumor progression of pancreatic ductal adenocarcinoma (PDAC) remains largely unexplored.

Given the critical dependency of KRas-driven tumors including PDAC on autophagy^4,7,8,25^, pharmacological or genetic inhibition of KRas-downstream RAF, MEK and ERK pathways has been shown to enhance autophagy, which supports the rationale for combining mitogen-activated protein (MAP) kinase inhibitors with a derivative of chloroquine (CQ), a lysosomotropic agent^26–28^. However, the therapeutic potential of CQ derivatives is limited by the requirement for high inhibitory doses and poor selectivity, highlighting the need for more potent and selective autophagy modulators.

Targeting ULK1, an autophagy-initiating kinase, has emerged as an alternative strategy for autophagy inhibition. A series of ULK1 inhibitors have demonstrated antitumor effects in vitro and in xenograft models^29,30^, and selective ULK1 inhibitors have shown synergistic tumor regression when combined with KRas or downstream effector inhibitors^31^. However, despite the increasing interest in ULK1-targeted therapies, the exact role of ULK1 in cancer progression remains poorly understood.

Here, we investigate how ULK1 function influences PDAC progression by using syngeneic orthotopic and spontaneous cancer GEM models. Our findings reveal that tumor-intrinsic ULK1 deletion suppresses PDAC progression by impairing autophagy-mediated tumor adaptation and by reprogramming the tumor microenvironment (TME) through altered infiltration of distinct immune cell subtypes, thereby providing tumor-suppressive immune states. These results provide insights into ULK1 as a promising therapeutic target in pancreatic cancer.

## Materials and methods

### Cell lines

MIA PaCa-2 and HEK293T cells were kindly provided by Drs. Yongdoo Choi and Jong Heon Kim (National Cancer Center Korea (NCC Korea)), which were originally purchased from the American Type Culture Collection (ATCC). KPC cell lines were a generous gift from Jong Heon Kim (NCC Korea) and were originally purchased from Ximbio; they were derived from PDAC tumors arising in KPC mice on a C57BL/6 background. All cells were cultured in Dulbecco’s Modified Eagle Medium (DMEM) supplemented with 10% fetal bovine serum (FBS; HyClone), 100 U/ml penicillin and 100 μg/ml streptomycin (Gibco) and were maintained at 37 °C in a humidified incubator with 5% CO_2_. For amino acid starvation media (-A.A), Earle’s Balanced Salt Solution (EBSS, HyClone) or Hank’s balanced saline solution (HBSS) was supplemented with 10% dialyzed FBS, glucose, vitamins, HEPES and minerals at the same concentrations as in DMEM.

### Generation of stable cell lines

CRISPR–Cas9-mediated KO of Ulk1 was performed with lentiCRISPR v2 vector (a gift from Feng Zhang; Addgene plasmid no. 52961; RRID no. Addgene_52961)^32^.

Single guide (sg)RNA oligo targeting mouse *Ulk1* CRISPR–Cas9 guide RNA (U0448BI200-1) or non-target sequence (sgControl) were synthesized (Gene Script). The guide RNA sequence was selected using the CRISPICK online tool (Broad institute, https://portals.broadinstitute.org/gppx/crispick/public) and sgRNA sequences are presented in Supplementary Table 2. Viral transduction processes were followed by standard protocols detailed in the Supplementary Materials and Methods.

### Reagents

Hoechst 33342 (H3570) was purchased from Thermo Fisher Scientific. Rapamycin (R0395) was purchased from Sigma-Aldrich.

### Cell proliferation and viability assay

Cell proliferation was monitored using the image-based cell proliferation analyzer IncuCyte (Essen Instruments). Cells were seeded in complete DMEM media and imaged throughout the indicated time period. An IncuCyte automated cell proliferation detector was used to measure cell confluence over time. Cell viability was determined by Annexin V and PI staining following standard protocols at the indicated time periods (556547; BD Biosciences), and analyzed using the FACS Verse analyzer (BD Biosciences). Viability was assessed on the basis of double-negative populations, and dead cells were defined by Annexin V^+^ and/or PI^+^ staining.

### Colony formation and spheroid cultures

KPC cells (5 × 102 cells per well) were seeded in 12-well plates in complete media (10% FBS in DMEM). After 7 days, cells were fixed for 10 min in 10% formalin (HT501128-4L; Sigma-Aldrich) and stained with 0.25% crystal violet (C6158; Sigma-Aldrich). For three-dimensional (3D) spheroids culture, KPC cells (1 × 103 cells per well) were seeded in 96-well round-bottom Ultra Low Attachment plates (7007; Corning) mixed with 1% Matrigel (354234; Corning) and complete media. Spheroid growth was monitored for 7 days and imaged using HCS system Operetta CLS (PerkinElmer). Quantification was performed by using Harmony software (PerkinElmer).

### Cell invasion assay

Invasion assay was performed to ascertain cell invasion using a 0.8-μm Transwell apparatus (Corning) with coated Matrigel (354234; Corning) on a 24-well plate. KPC cells (1 × 10^5^) suspended in 100 μl serum-free medium were seeded into the upper chamber and complete DMEM was added to the lower chamber. After incubation at 37 °C for 24 h, invaded cells were stained using Diff-Quik reagents (Sysmex Corporation) and counted under an inverted microscope at a 4× and 20× magnifications.

### LSL-Kras^G12D/+^; LSL-Trp53^R172H/+^; Pdx1-Cre;Ulk1^fl/fl^ GEM models

*Kras*^*G12D*/+^ (B6.129S4-*Kras*^*tm4Tyj*^, strain no. 01XJ6) was received from the National Cancer Institute (NCI) mouse repository. *Trp53*^*R172H*/+^ (129S-*Trp53*^*tm2Tyj*^/J, strain no. 008652) and *Pdx1*-Cre (B6.FVB-Tg (*Pdx1*-Cre)6Tuv/J, strain no. 014647) mice were purchased from the Jackson Laboratory. These mice were backcrossed more than six times with C57BL/6 and were subsequently used for generating KPC (*Kras*^*G12D*/+^; *Trp53*^*R172H*/+^; *Pdx1*-Cre) mice. The KPC model of PDAC was first described in 2005 and incorporates, through Cre-lox technology, the conditional activation of mutant endogenous alleles of the *Kras* and *Trp53* gene^33^. *Ulk1*^fl/fl^ (B6.129-*Ulk1*^*tm1Thsn*^/J, stock no. 017976) mice were purchased from The Jackson Laboratory, which were generated and donated by Craig B. Thompson (Memorial Sloan Kettering Cancer Center)^24^. The *Ulk1*^*fl/fl*^ and KPC mice were crossed, and after several rounds of breeding, *KPC*;*Ulk1*^*fl/fl*^ mice were successfully obtained. All animal procedures were performed in accordance with a protocol approved by the Institutional Animal Care and Use Committee (IACUC) of the National Cancer Center (NCC). The NCC is an Association for Assessment and Accreditation of Laboratory Animal Care International-accredited facility that abides by the guidelines of the Institute of Laboratory Animal Resources Guide and Usage Committee. The methods applied in this study were performed in accordance with the approved guidelines.

### Orthotopic pancreatic cancer mouse model

Here, 6-week-old female C57BL/6 mice (Orient Bio) were orthotopically injected with 2 × 10^5^ KPC sgSC or KPC sg*Ulk1* cells suspended in 1:1 Matrigel (50 µl) into the pancreatic parenchyma. After injection, the peritoneum and skin were closed with a 6-0 suture (639 G; Ethicon). A total of 3 weeks after the allograft, the pancreas was isolated from the mice and used for the indicated experiments.

### Histological and IHC analyses

Pancreatic tissues from indicated orthotopically transplanted mice or *KPC*;*Ulk1*^+/*fl*^ and *KPC*;*Ulk1*^*fl/fl*^ GEM mice were isolated from 12–17-week-old mice, respectively. The tissues were fixed in 10% neutral buffered formalin (BN019, Biosolution) and paraffin embedded. Sections (4 μm thick) of mouse pancreas were deparaffinized, rehydrated and incubated in boiling CC1(pH 9) or CC2(pH 6) for antigen retrieval by Benchmark TX (950-123 or 124; Ventana Medical Systems) or Discovery XT (Ventana Medical Systems).

Immunohistochemical (IHC) staining was performed with the indicated primary antibodies (Supplementary Table 1) and then detected with a DAB detection kit (Ventana Medical Systems) according to the manufacturer’s instructions, followed by counterstaining with hematoxylin (Ventana Medical Systems). The stained images were acquired using the Vectra Polaris (PerkinElmer/Akoya Biosciences) and quantified using inForm Tissue Analysis Software (Akoya Biosciences). We calculated the H-score as follows: H-Score = (1 × % of 1+ staining spot) + (2 × % of 2+ staining spot) + (3 × % of 3+ staining spot). Statistical analyses were performed using Graphpad Prism 8.0.1.

### Multi-IHC assay

Tissue microarray (TMA) sections from patients with PDAC (PA241e, TissueArray.Com) and the formalin-fixed paraffin-embedded (FFPE) pancreatic tissue from KPC *Ulk1*^+/*fl*^ and KPC *Ulk1*^*fl/fl*^ mice were subjected to multiplex immunohistochemistry (multi-IHC) with indicated antibodies (Supplementary Table 1) and automated staining Leica Bond RX (Leica Biosystems) using an Opal 7 kit (nos. NEL871001KT and ARR1001KT; Akoya Biosciences). Tyramide signal amplification fluorophores were used to combine with each antibody separately according to manufacturer’s instructions. Imaging and quantification were performed by Vectra Polaris Automated Quantitative Pathology Imaging System (PerkinElmer) and inForm image analysis software (Akoya Biosciences). Data were compiled and analyzed after merging and consolidating each case by R studio (version 2021.09.2.0).

### Flow cytometry for tumor immunophenotyping

For tumor immunophenotyping, tumor tissues were dissociated in a fully automated way by using the gentleMACS Octo Dissociator with Heaters (130-096-427; Miltenyi Biotec) with collagenase P (C7657, Merck) and the Tumor Dissociation Kit (130-096-730; Miltenyi Biotec), which are optimized for epitope preservation. Digested tissues were filtered through a 70-μm nylon cell strainer (352350; BD Falcon). Cells were blocked with antimouse CD16/CD32 antibody (Mouse BD Fc Block) (BD553142, clone 2.4G2; BD Biosciences) and stained with the indicated antibodies for 20 min at 4 °C. Intracellular staining was performed using a Fixation/Permeabilization Kit (BD554714; BD Bioscience). Samples were analyzed on a LSRFortessa Cell Analyzer (BD Biosciences).

### Cytokine array

Cytokine profiles were assessed using the Proteome Profiler Mouse Cytokine Array Kit (ARY006; R&D systems) following the manufacturer’s instruction. Detailedly, mouse pancreatic cancer tissues were homogenized by Tissue Lyser II (QIAGEN) in PBS containing protease inhibitor cocktail (Roche) and then lysed with 1% Triton X-100. Cellular debris were removed by centrifugation at 10,000*g* for 5 min at 4 °C, and protein was quantified using BCA Protein Assay (23227; Thermo Fisher Scientific). Conditioned media (CM) were collected from cells cultured under identical conditions for 24 h. Media were then normalized to the total cellular protein content, as determined from corresponding cell lysates. Membranes incubated with CM were developed and visualized using the FUSION SOLO S imaging system (Vilber) with exposure times ranging from 2 to 6 min.

### Immune cell viability analysis

Pancreas, spleen and blood from the abdominal aorta were collected and dissociated as indicated above. Polymorphonuclear myeloid-derived suppressor cells (PMN-MDSC)/neutrophils (CD45^+^CD11b^+^Ly6G^+^), CD8^+^ T cells (CD45^+^CD3^+^CD8^+^) and dendritic cells (DCs) (CD45^+^CD11c^+^) were sorted using Melody (BD Biosciences) and then cultured in the presence of 50% CM from KPC sgSC or KPC_sg*Ulk1* cells on a 384- or 96-well black plate (6057308 and 6055302; PerkinElmer). After incubation for the indicated time, live cell numbers were assessed using calcein-AM (C1430; Invitrogen) staining. Imaging and quantification were performed using the Operetta CLS system and Harmony software (PerkinElmer).

### Bioinformatic data processing

The Cancer Genome Atlas (TCGA)-pancreatic adenocarcinoma (PAAD) patients were stratified with the autophagy score using the surv_cutpoint function from the survminer package (version 0.4.9). Kaplan–Meier overall survival plots and the *P* value between patient groups were calculated using the log-rank test implemented in the survival package (version 3.5-7). Cox hazard ratios and confidence intervals were calculated using the coxph function of the survival package.

The autophagy score was calculated using a modified version of ssGSEA2.0, which is an adaptation of the GSEA algorithm for single-cell analysis^34,35^. The list of genes used for the autophagy score calculation is provided in the Supplementary Information. Default parameters for ssGSEA were used to calculate scores for TCGA-PAAD patients and for cells in single-cell data from patients with pancreatic cancer (CRA001160)^35,36^. To stratify patients into high and low autophagy groups, we used the surv_cutpoint function from the survminer package in R. This method identifies the optimal threshold that best separates patient groups on the basis of survival outcome. The resulting cutoff value was 6.975026, with patients scoring greater than or equal to this value classified as ‘high autophagy’.

### Patient filtering and cohort size

We initially downloaded 183 PAAD samples directly from the TCGA_PAAD cohort, as described in the Methods. Of these, 178 patients had valid overall survival information and 162 patients had both survival information and complete somatic mutation single-nucleotide variant (SNV) profiles.

For the survival analyses, we included only those patients with sufficient clinical metadata. Specifically, the 162 patients with both survival data and complete mutation profiles were used in cases where mutation-based subgrouping or stratification was considered.

### Statistical analysis

Immunoblot quantifications were performed using ImageJ software version 1.50i (NIH). Statistical significance was calculated using Student’s *t*-test in GraphPad Prism 8. *P* < 0.05 was considered statistically significant (^*^P < 0.05; ^**^P < 0.01; ^***^P < 0.001). Data are expressed as standard error of the mean (s.e.m.), which are from at least three independent experiments.

## Results

### ULK1 activity is elevated in pancreatic cancer independent of mRNA expression

To assess the clinical relevance of autophagy proteins in pancreatic cancer, we analyzed the expression of 25 core autophagy proteins in PAAD from TCGA. These autophagy gene sets were significantly upregulated in tumor tissues compared with adjacent normal tissues (*P* < 0.05; Supplementary Fig. 1a–c), and high expression levels of autophagy gene sets correlated with poor 5-year overall survival (Supplementary Fig. 1d), supporting the tumor-promoting role of autophagy in PDAC.

**Fig. 1:**
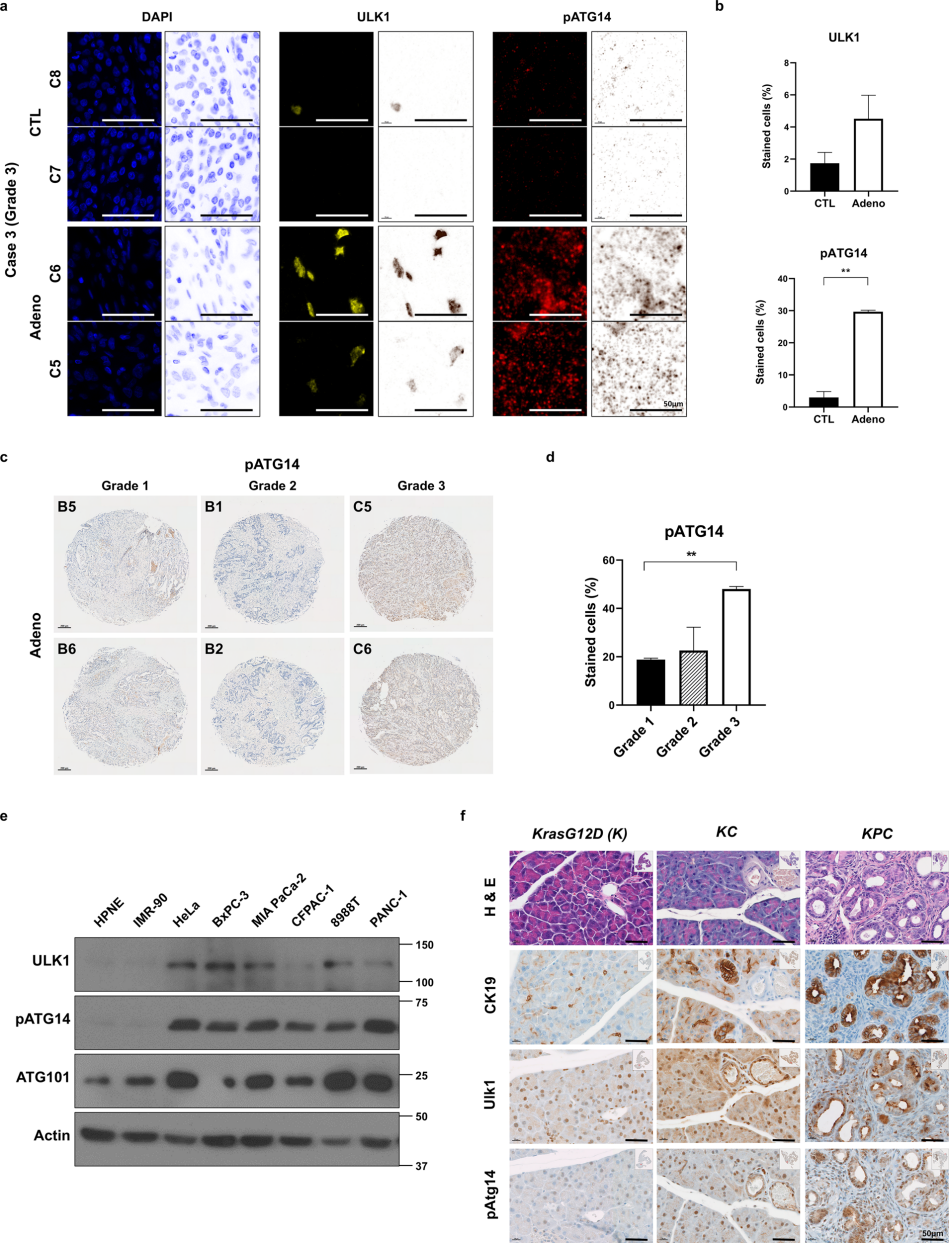
ULK1 expression and activities are upregulated in pancreatic cancers. **a**,**b** Representative images (**a**) and quantitative data (**b**) of ULK1 (yellow) and pATG14 (red) in human PAAD TMA using multi-IHC. Tissues from six patients were analyzed, each including matched adjacent normal and tumor tissue (four cores per patient). Images from representative grade 3 PAAD cases are shown. C7 and C8 cores indicate adjacent normal pancreas tissue (control (CTL)), while C5 and C6 represent adenocarcinoma (Adeno) tumor regions. Scale bars are indicated in the figures. Error bars indicate the mean ± s.e.m. from two independent cores (ULK1 *P* = 0.2295; pATG14 *P* = 0.0050). **c**,**d** Representative images (**c**) and quantification (**d**) of pATG14 by grade using IHC staining in adenocarcinoma cores from human PAAD TMA, stratified by tumor grade (grade 1 versus 3 *P* = 0.0016). **e** Immunoblot analysis of ULK1, pATG14, ATG101 and β-Actin (loading control) in human pancreatic cancer cell lines. **f** Representative images of hematoxylin and eosin (H&E) staining and IHC staining for CK19, Ulk1 and pAtg14 in pancreas tissue from K, KC and KPC mice. Scale bars are indicated in the figures. All values were considered statistically significant by Student’s *t*-test (^*^P < 0.05; ^**^P < 0.01).

Interestingly, the messenger RNA level of *ULK1* was not significantly different between tumor and normal tissues (Supplementary Fig. 1e). However, multi-IHC revealed elevated ULK1 protein levels and activity, marked by phosphorylated ATG14 (pATG14), particularly in high-grade (grade 3) pancreatic tumors compared with normal tissues (Fig. 1a–d and Supplementary Fig. 2a,b).

Similarly, increases in ULK1 and pATG14 levels were observed in human pancreatic cancer cell lines compared with normal human pancreatic epithelial and fibroblast (IMR90) cells (Fig. 1e). Moreover, elevated expression of ULK1 and pATG14 was detected in transformed pancreatic intraepithelial neoplasia in *Kras*^+/*LSL-G12D*^; *Pdx1*-Cre (KC) mice and in advanced PDAC lesions from *Kras*^+/*LSL-G12D*^; *Trp53*^*R172H*/+^; *Pdx1*-Cre (KPC) mice (Fig. 1f). These results suggest that ULK1 activity rather than its mRNA levels is markedly elevated in malignant pancreatic cancers, supporting its potential role in tumor progression.

### ULK1 depletion suppresses pancreatic cancer cell proliferation, invasion and autophagy

**Fig. 2:**
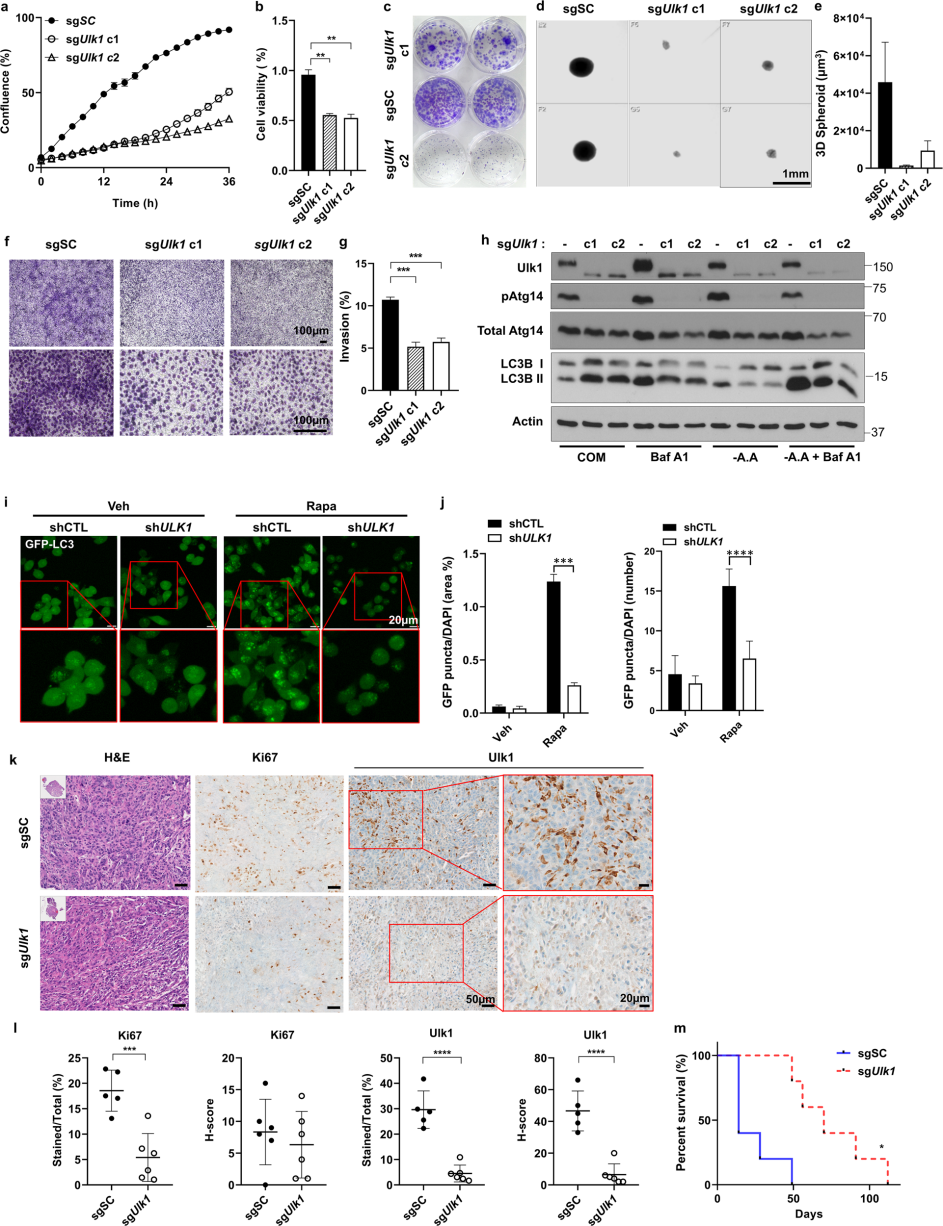
Ulk1 regulates tumorigenesis in an orthotopic pancreatic cancer model. **a** Relative cell proliferation between sgSC, sg*Ulk1* KPC clone 1 (c1) and clone 2 (c2) cells analyzed by IncuCyte over 48 h (*P* < 0.0001 for all time points after 2 h). **b** Relative cell viability between sgSC, sg*Ulk1* c1 and c2 KPC cells analyzed by MTT assay over 72 h (sgSC versus sg*Ulk1* c1 *P* = 0.0015, sgSC versus sg*Ulk1* c2 *P* = 0.0022). **c** The colony formation assays using sgSC, sg*Ulk1* c1 and c2 KPC cells. **d**,**e** Bright field images (**d**) and quantification (**e**) showing 3D spheroid formation using sgSC, sg*Ulk1* c1 and c2 KPC cells. Image analysis and quantification were performed using the ImageJ program (sgSC versus sg*Ulk1* c1 *P* = 0.1049, sgSC versus sg*Ulk1* c2 *P* = 0.1705). **f**,**g** Representative images captured at 4× and 20× magnification (**f**) and quantification (**g**) of Transwell migration and Matrigel invasion assays analyzed using sgSC, sg*Ulk1* c1 and c2 KPC cells (sgSC versus sg*Ulk1* c1 *P* = 0.0001, sgSC versus sg*Ulk1* c2 *P* = 0.0001). (**h**) Immunoblot analysis of Ulk1, pAtg14, Atg14, LC3 and β-Actin (loading control) in sgSC or sg*Ulk1* KPC cells cultured in complete medium (COM) or replaced by amino acid-deprived media for 2 h with or without 100 nM bafilomycin A1. **i**,**j**, Representative images of GFP-LC3 puncta (**i**) and quantification (**j**) in MIA PaCa-2 cell line harboring shControl (shCTL) or sh*ULK1* after treatment with either 4 µM of rapamycin (Rapa) or vehicle (Veh) for 4 h (area, *P* = 0.0002; number, *P* < 0.0001). **k**,**l** Representative images (**k**) and quantitative data (**l**) of H&E staining and Ki67 and Ulk1 by IHC in pancreatic tissues obtained from orthotopic models of PDAC injected by KPC sgSC or KPC sg*Ulk1* (Ki67 *P* = 0.0008/H-score *P* = 0.5206, Ulk1 *P* < 0.0001/H-score *P* < 0.0001). (**m**) Survival graph illustrating orthotopic mouse models of PDAC, with each group consisting of *n* = 5 mice (*P* = 0.0214). The models were generated using KPC cells carrying either sgSC or sg*Ulk1*, and the observation period extended up to 119 days. Scale bars are indicated in the figures, and error bars indicate the mean ± s.e.m. for three independent images. All values were considered statistically significant by Student’s *t*-test (^*^P < 0.05; ^**^P < 0.01; ^***^P < 0.001; ^****^P < 0.0001).

**Fig. 3:**
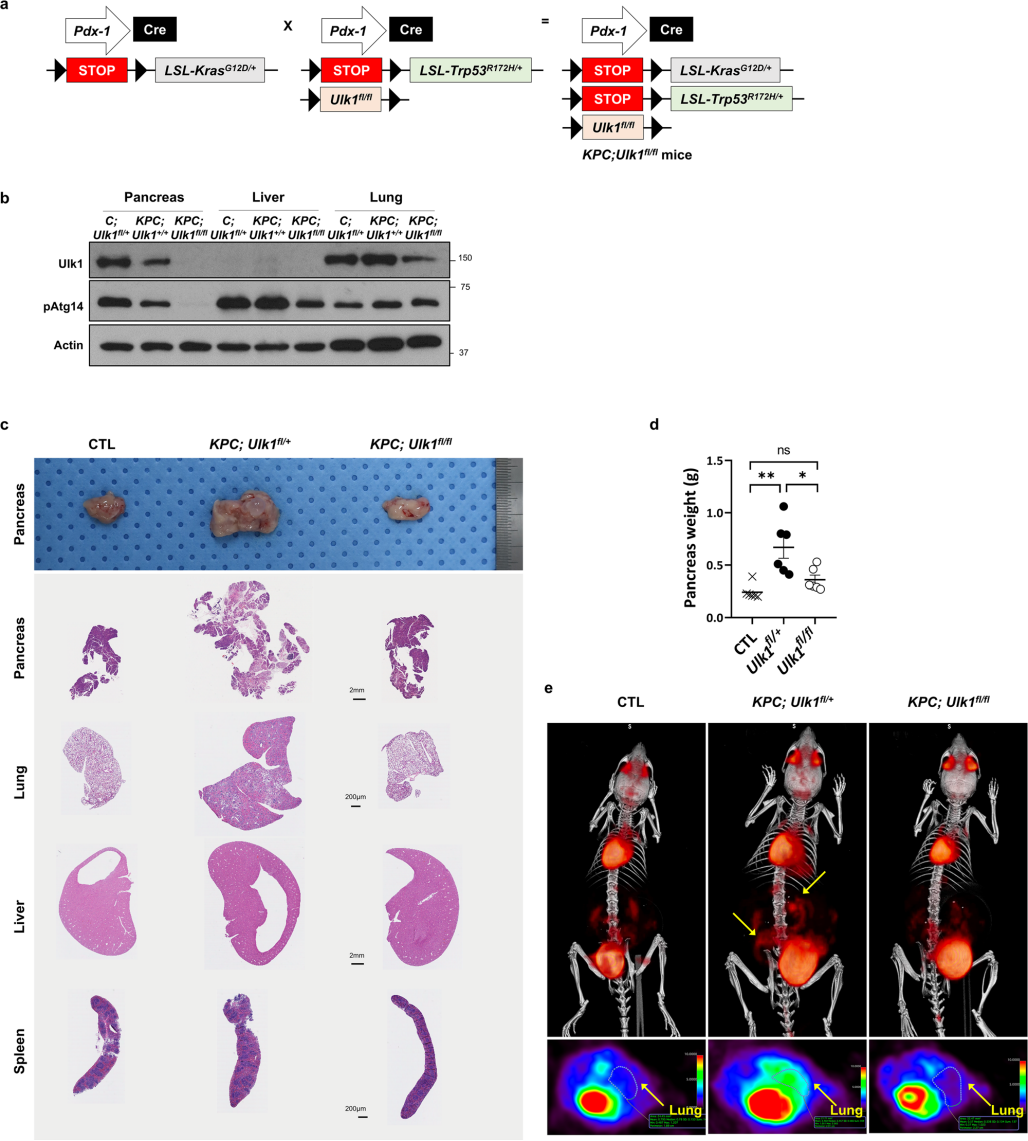
Pancreas-specific *Ulk1* deletion suppresses pancreatic cancer development in KPC GEM model. **a** Breeding strategy for generation of *KPC;Ulk1*^*fl/fl*^ mice. **b** Immunoblot analysis of ULK1, pATG14 and β-Actin (loading control) in the indicated organs of *KPC;Ulk1*^*fl/fl*^ mice compared with controls (*C;Ulk1*^*fl*/+^ or *KPC;Ulk1*^+/+^). **c**, Macroscopic pancreas morphology (top) and H&E staining (bottom) of various organs from normal CTL, *KPC;Ulk1*^*fl*/+^ and *KPC;Ulk1*^*fl/fl*^ mice. **d** Quantification of pancreas weight (*n* = 6; *P* = 0.0207). Error bars indicate the mean ± s.e.m. **e** Positron emission tomography–computed tomography imaging showing tumors indicated by arrows from CTL, *KPC;Ulk1*^*fl*/+^ and *KPC;Ulk1*^*fl/fl*^ mice.

**Fig. 4:**
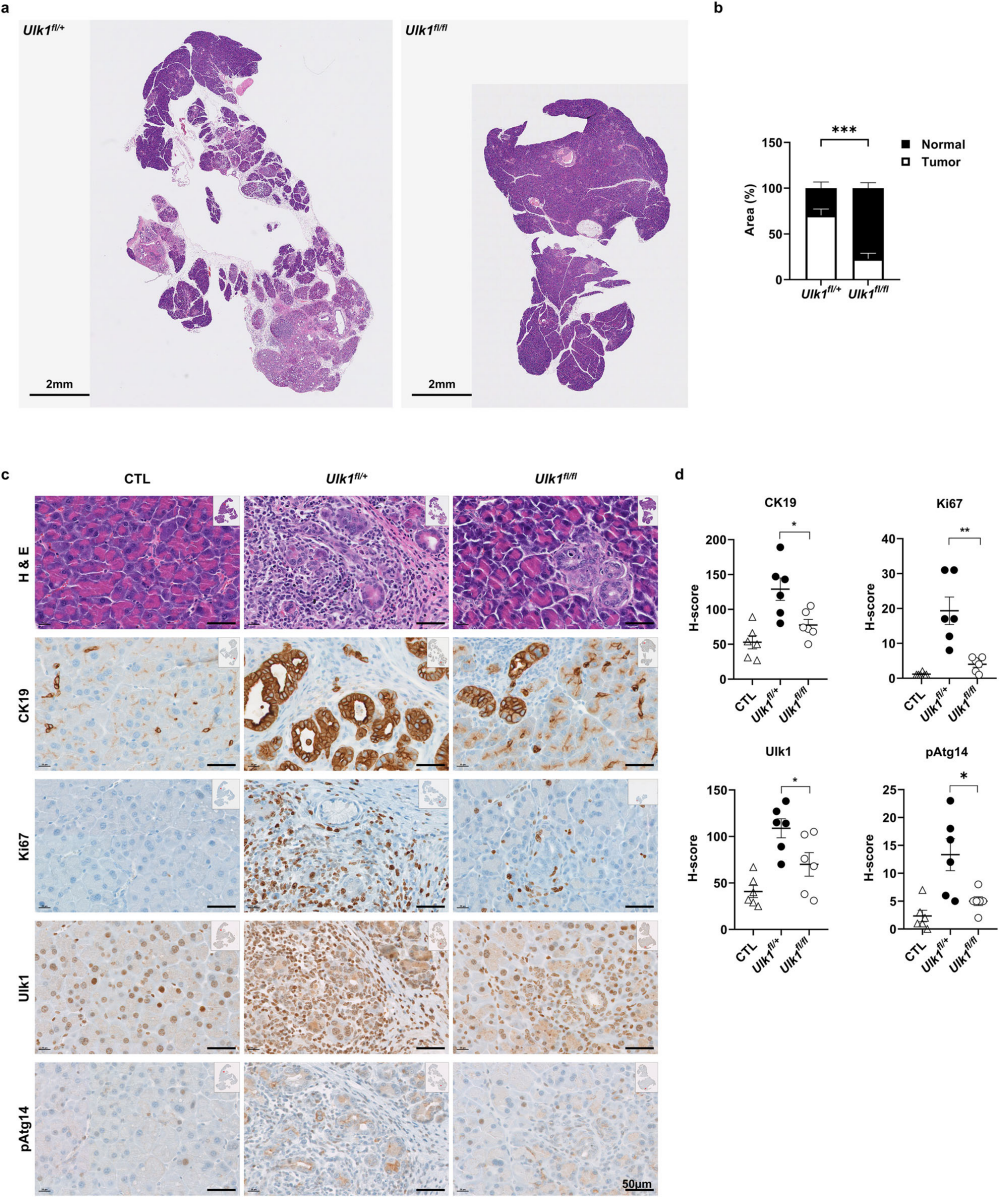

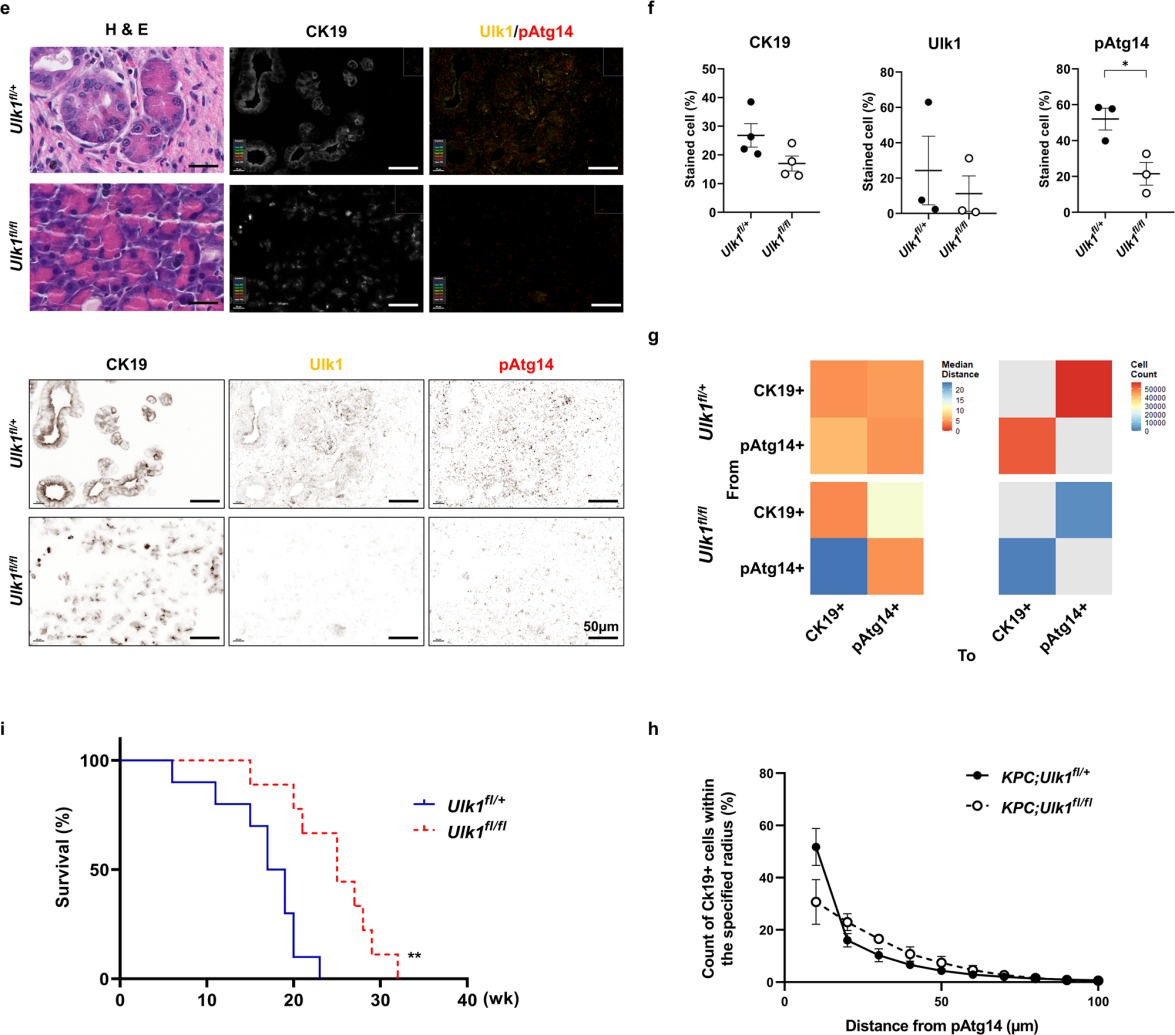
Ulk1 activity is associated with pancreatic tumor transformation in KPC mice. **a**,**b** Representative images of H&E staining (**a**) and quantification of tumor area (**b**) in pancreatic tissues from *KPC;Ulk1*^*fl*/+^ and *KPC;Ulk1*^*fl/fl*^ GEM mice (*n* = 6; *P* = 0.0087). Error bars indicate the mean ± s.e.m. for six independent experiments (*P* = 0.0087). **c**,**d** Representative images (**c**) and quantitative data (**d**) of H&E staining and CK19, Ki67, Ulk1 and pAtg14 by IHC in pancreas tissues from *KPC;Ulk1*^*fl*/+^ and *KPC;Ulk1*^*fl/fl*^ mice (CK19 H-score *P* = 0.017, Ki67 H-score *P* = 0.0035, Ulk1 H-score *P* = 0.0386 and pAtg14 H-score *P* = 0.0187). **e**,**f** Multi-IHC images (**e**) and quantification (**f**) for H&E, CK19 (white), Ulk1 (yellow) and pAtg14 (red) in pancreas tissues from *KPC;Ulk1*^*fl*/+^ and *KPC;Ulk1*^*fl/fl*^ mice (CK19 *P* = 0.0898, Ulk1 *P* = 0.5825, pATG14 *P* = 0.0255). Scale bars are indicated in the figures, and error bars indicate the mean ± s.e.m. for over four independent experiments. **g**,**h** Spatial analysis of CK19^+^ and pAtg14^+^ cells with quantification of their proximity in tumor tissues. Heat map showing median distance (**g**, left) and cell count (**g**, right) and quantitative data (**h**) representing pAtg14+ cells near or far from CK19+ malignant cell phenotypes across the mouse cohort. Error bars indicate the mean ± s.e.m. for three independent experiments. (at 10 µm, *P* = 0.1313). **i**, Kaplan–Meier survival analysis of *KPC;Ulk1*^*fl*/+^ (*n* = 10) and *KPC;Ulk1*^*fl/fl*^ (*n* = 9) mice (*P* = 0.0011). All values were considered statistically significant by Student’s *t*-test (^*^P < 0.05; ^**^P < 0.01; ^***^P < 0.001; ^****^P < 0.0001).

To investigate the functional role of ULK1 in pancreatic cancer progression, we generated *Ulk1* KO KPC cells using CRISPR–Cas9, which was confirmed by genomic sequencing and quantitative PCR with reverse transcription (Supplementary Fig. 3a,b). *Ulk1* KO cells exhibited significantly impaired cell growth compared with control cells, as measured by IncuCyte live-cell imaging (Fig. 2a) and 3-(4,5-dimethylthiazol-2-yl)-2,5-diphenyltetrazolium bromide (MTT) assay (Fig. 2b). ULK1 loss also reduced colony formation (Fig. 2c) and markedly impaired 3D spheroid growth (Fig. 2d,e). In addition, Transwell invasion assay showed a substantial decrease in invasion of *Ulk1* KO cells compared with sgScrambled (sgSC) control (Fig. 2f,g), indicating a significant reduction in both proliferative and invasive potential.

To determine whether these phenotypes were linked to autophagy, we assessed autophagic flux by analyzing LC3 processing and localization. Compared with *Ulk1* WT cells, *Ulk1*-deficient cells showed LC3-I accumulation and reduced conversion to LC3-II, particularly under amino acid-deprived conditions. This was further supported by a significantly lower LC3-II:I ratio in bafilomycin A1-treated cells, indicating defective autophagic flux upon Ulk1 deletion (Fig. 2h and Supplementary Fig. 3c). In addition to the reduced LC3-II conversion rate, the absolute LC3-II levels, normalized by loading control, were also significantly lower in *Ulk1*-deficient cells even with the treatment of bafilomycin A1 (Fig. 2h and Supplementary Fig. 3c). Rapamycin-induced GFP-LC3 puncta formation was also significantly impaired in ULK1 KO human pancreatic cancer cells (Fig. 2i,j), further confirming autophagy suppression and defect in viability (Supplementary Fig. 3d). These findings indicate that ULK1 is required for nutrient-responsive autophagy and that its depletion impairs both the growth and invasion of pancreatic cancer cells.

### ULK1 loss delays tumor progression in orthotopic pancreatic tumor model

To extend our in vitro findings, we investigated the in vivo role of ULK1 in tumor progression using a syngeneic mouse model. An orthotopic PDAC mouse model was established by injecting *Ulk1* KO (sg*Ulk1*) or control (sgSC) KPC cells into the pancreas of C57BL/6J mice. Histological analysis 3–5 weeks after injection revealed reduced Ki67 staining in *Ulk1*-deficient tumors compared with controls, consistent with decreased Ulk1 expression (Fig. 2k,l). Moreover, mice bearing *Ulk1* KO tumors showed significantly extended survival compared with control KPC tumor-injecting mice (Fig. 2m). These findings support a critical role of Ulk1 in promoting cell proliferation and tumor progression in anorthotopically transplanted in vivo model.

### Ulk1 deficiency suppresses pancreatic tumor development in a spontaneous mouse model

To further investigate the role of ULK1 in pancreatic cancer progression, we used the *LSL-Kras*^*G12D*/+^*; LSL-Trp53* ^*R172H*/+^*; Pdx1-Cre* (KPC) mouse model^33^, which spontaneously develops pancreatic tumors that mimic the pathology of human PDAC. These mice were crossbred with previously established *Ulk1*^*fl/fl*^ mice^24^ to generate *KPC;Ulk1*^*fl/fl*^ mice, enabling pancreas-specific *Ulk1* deletion via Cre recombinase (Fig. 3a). In this GEM model, the pancreas-specific expression of Cre recombinase, driven by *Pdx1*-Cre, induces a constitutively active *Kras* mutation (G12D) and a dominant negative *Trp53* mutation (R172H) (Supplementary Fig. 4a). Immunoblotting confirmed efficient Cre-mediated *Ulk1* deletion in pancreatic tissues (Fig. 3b). Histological analysis showed only crucial abnormalities in pancreas rather than other tissues, although occasional lung pathology was observed in positive control (*KPC*;*Ulk1*^*fl*/+^) mice, suggesting that *Ulk1* deletion specifically impacts pancreatic tumor development (Fig. 3c and Supplementary Fig. 4b). However, pancreas weights were significantly reduced in *KPC*;*Ulk1*^*fl/fl*^ mice compared with that of KPC control mice (*KPC*;*Ulk1*^*fl*/+^) (Fig. 3d). Moreover, 18F-fluorodeoxyglucose positron emission tomography–computed tomography (18F-FDG-PET-CT) imaging revealed a reduced tumor burden and lower maximum standardized uptake values (SUVmax) in *KPC*;*Ulk1*^*fl/fl*^ mice, compared with *KPC*;*Ulk1*^*fl*/+^ control mice (Fig. 3e and Supplementary Fig. 4c). Notably, increased fluorodeoxyglucose (FDG) uptake in the lungs, indicative of distant metastasis, was observed in *KPC*;*Ulk1*^*fl*/+^ control mice but diminished in *KPC*;*Ulk1*^*fl/fl*^ mice. These findings suggest that *Ulk1* deletion primarily impacts pancreatic tumor development in the KPC model, with occasional lung metastasis in the late stages of cancer.

Next, further histopathological results from the pancreas showed that all KPC control mice (*KPC*;*Ulk1*^*fl*/+^ or *KPC*;*Ulk1*^+/+^) developed moderate-to-malignant PDAC within 18 weeks, whereas only 12% (1/8) of *KPC*;*Ulk1*^*fl/fl*^ mice (pancreas-specific *Ulk1* KO KPC) developed low-grade pancreatic intraepithelial neoplasia lesions, with the majority displaying normal pancreatic architecture with healthy acini cells (Fig. 4a,b). IHC staining further revealed significantly lower levels for cytokeratin 19 (CK19) and Ki67 in *KPC*;*Ulk1*^*fl/fl*^ pancreatic tissues compared with those from KPC control (*KPC*;*Ulk1*^*fl*/+^) mice, consistent with suppressed epithelial transformation and proliferative capacity (Fig. 4c,d). These findings aligned with reduced expression of Ulk1 and pAtg14 in the pancreatic ductal regions of *KPC*;*Ulk1*^*fl/fl*^ mice, which was more pronounced in the transformed regions of control KPC mice (Fig. 4c,d). In agreement with impaired autophagic flux, LC3B, an autophagy marker, was significantly decreased in the pancreas of *KPC*;*Ulk1*^*fl/fl*^ mice (Supplementary Fig. 4d,e).

Multi-IHC further confirmed that Ulk1 and pAtg14 levels were significantly enriched in CK19^+^ epithelial cells within tumors from *KPC*;*Ulk1*^*fl*/+^ mice, while these signals were nearly absent in the tissue from *KPC*;*Ulk1*^*fl/fl*^ mice (Fig. 4e,f). Spatial analysis supported a strong association between CK19^+^ adenocarcinoma cells in and pAtg14^+^ autophagy-active cells in *KPC*;*Ulk1*^*fl*/+^ tumors, which was largely diminished in Ulk1-deficient mice (Fig. 4g,h). Importantly, *KPC*;*Ulk1*^*fl/fl*^ mice exhibited a significantly prolonged median overall survival—approximately 10 weeks longer than that of *KPC*;*Ulk1*^*fl*/+^ controls (Fig. 4i). Collectively, these data demonstrate that Ulk1 is critical for PDAC progression in a physiologically relevant spontaneous PDAC model and support its function as a key effector of both primary tumor growth and metastatic potential.

### Ulk1 KO alters tumor immune microenvironment and enhances antitumor immunity

**Fig. 5:**
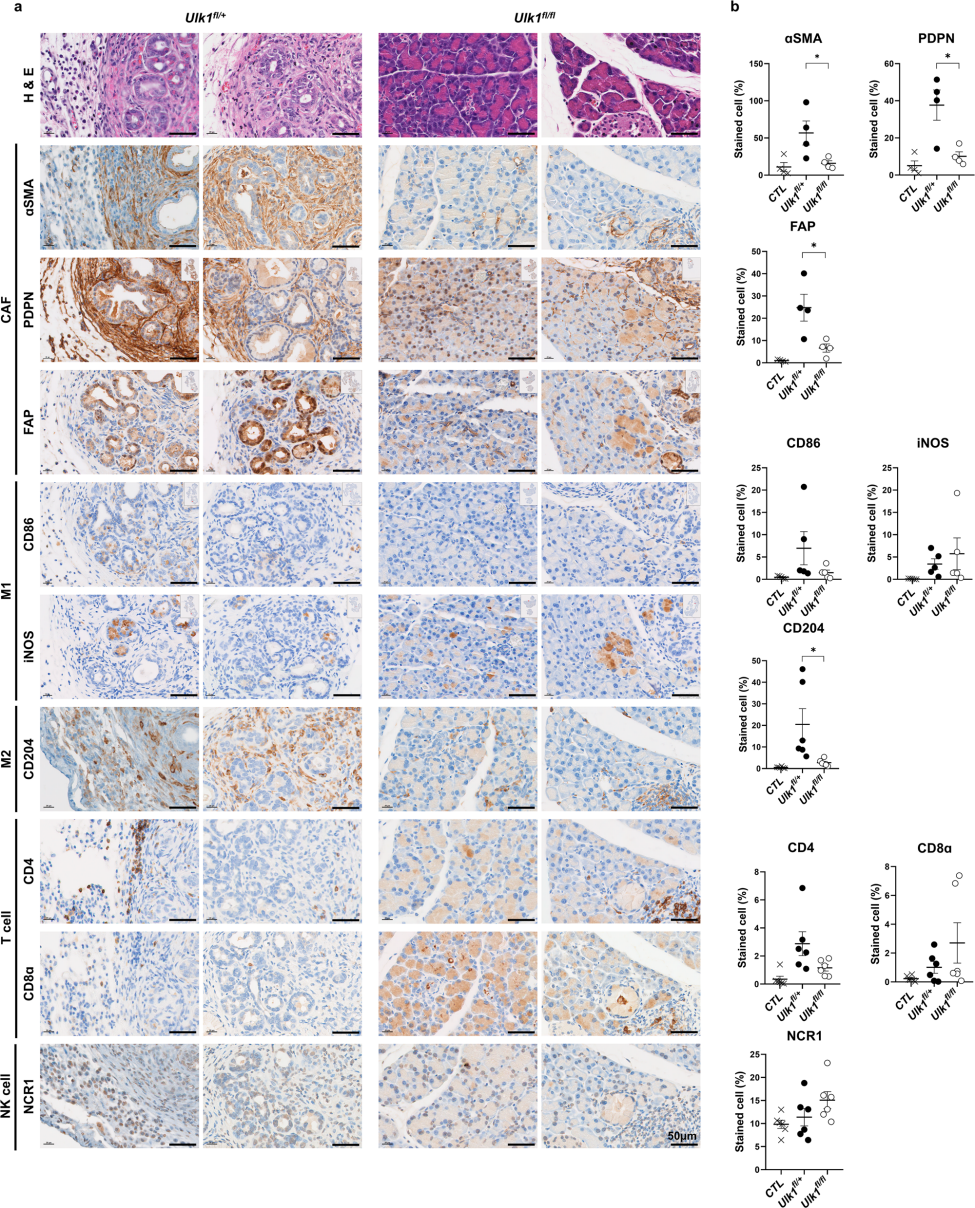
Pancreas-specific *Ulk1* depletion alters tumor immune microenvironment in KPC mice. **a**,**b** Representative images (**a**) and quantitative data (**b**) of H&E staining and αSMA, PDPN, FAP, CD86, iNOS, CD204, CD4, CD8α and NCR1 in pancreas tissue from *KPC*;*Ulk1*^*fl*/+^ and *KPC*;*Ulk1*^*fl/fl*^ GEM mice. Scale bars are indicated in the figures, and error bars indicate the mean ± s.e.m. for over four independent experiments (αSMA *P* = 0.0486, PDPN *P* = 0.0296, FAP *P* = 0.0421, CD86 *P* = 0.2302, iNOS *P* = 0.4515, CD204 *P* = 0.0350, CD4 *P* = 0.0791, CD8α *P* = 0.2718 and NCR1 *P* = 0.1920). All values were considered statistically significant by Student’s *t*-test (^*^P < 0.05; ^**^P < 0.01; ^***^P < 0.001; ^****^P < 0.0001).

Given the role of autophagy in modulating tumor immunity^37,38^, we next examined whether *Ulk1* deletion alters immune cell composition within the TME. IHC analysis revealed that cancer-associated fibroblast (CAF) markers (αSMA, PDPN and FAP) and immunosuppressive M2 macrophage marker CD204 were more abundant in tumors from *KPC*;*Ulk1*^*fl*/+^ control mice, particularly in CK19^+^ adenocarcinoma lesions, whereas these markers were notably reduced in the pancreas of *KPC*;*Ulk1*^*fl/fl*^ mice (Fig. 5a,b). By contrast, cytotoxic T cell (CD8α) and natural killer (NK) cell (NCR1) staining tended to be substantially higher in *Ulk1* KO tumors compared with controls, suggesting a possible shift toward a more antitumor immune profile. M1 macrophage markers (CD86 and iNOS) and CD4^+^ T cell levels did not differ significantly between groups (Fig. 5a,b).

Multi-IHC further confirmed that, CD204^+^ M2 macrophages were more abundant in control tumor (*KPC*;*Ulk1*^*fl*/+^) mice, while CD8^+^ T cells were substantially more enriched in the pancreas of *KPC*;*Ulk1*^*fl/fl*^ mice than in control KPC mice, although the results were not statistically significant (Supplementary Fig. 5a–c).

Collectively, these findings indicate that tumor *Ulk1* deletion remodels the pancreatic TME primarily by reducing immunosuppressive immune cell populations, along with a modest increase in cytotoxic lymphocyte infiltration.

### ULK1 deletion reshapes immune cell composition in pancreatic tumors

To elucidate the molecular mechanisms underlying tumor regression following *Ulk1* depletion, we performed RNA-sequencing analysis of *Ulk1* WT and *Ulk1* KO KPC cells. Differential expression gene analysis identified 4,097 genes with altered expression, including 1,071 upregulated and 1,538 downregulated genes in *Ulk1* KO cells, visualized by heat maps and volcano plots (Supplementary Fig. 6 and Supplementary Table 6). Hierarchical clustering of differentially expressed genes showed distinct profiles between the genotypes under both amino acid-complete (com) and -deprived conditions (-A.A). (Supplementary Fig. 6a). Volcano plots were also visualized using log_2_ fold changes (log_2_FC| > 2), and *P* values (*P* < 0.05) derived from comparisons between WT-com and KO-com (Supplementary Fig. 6b) Kyoto Encyclopedia of Genes and Genomes (KEGG) pathway enrichment analysis of upregulated gene sets in *Ulk1* KO cells revealed significant enrichment in ‘MAP kinase signaling’, ‘PI3K–Akt signaling’ and ‘cytokine–cytokine receptor interaction’ pathways (Supplementary Table 3). Among these pathways, the gene set of cytokine–cytokine interaction pathway upregulated in *Ulk1* KO cells was indicated in the volcano plot (Supplementary Fig. 6b).

In addition, proteomic profiling of *KRas*^*G12D*^-expressing human pancreatic epithelial cells treated with CQ revealed a similar upregulation of immune-related pathways, with ‘cytokine–cytokine receptor interaction’, ‘JAK–STAT signaling’, ‘TGF-β signaling’ and ‘mitophagy’ among the most enriched pathways (Supplementary Table 4).

Complementing these results, gene set enrichment analysis (GSEA) of TCGA pancreatic cancer datasets^34^ revealed that lower *ULK1* expression correlated with elevated immune and mitochondrial gene signatures. Gene ontology analysis of gene clusters negatively co-expressed with *ULK1* revealed strong enrichment for terms related to ‘antigen processing and presentation’, ‘immune system processes’ and ‘mitochondrial metabolism’, suggesting a link between *ULK1* suppression and activation of immune and mitochondria metabolic pathways (Supplementary Table 5).

To further assess whether tumor-intrinsic *Ulk1* deletion affects immune cell composition in vivo, we used syngeneic orthotopic models by injecting KPC sgSC (*Ulk1* WT) and KPC sg*Ulk1* (*Ulk1* KO) cells into the pancreas of immunocompetent C57BL/6J mice. After 3 weeks, the mice were killed and subjected to immunophenotyping by fluorescence-activated cell sorting (FACS) analysis using the gating strategy shown in Supplementary Fig. 7.

**Fig. 6:**
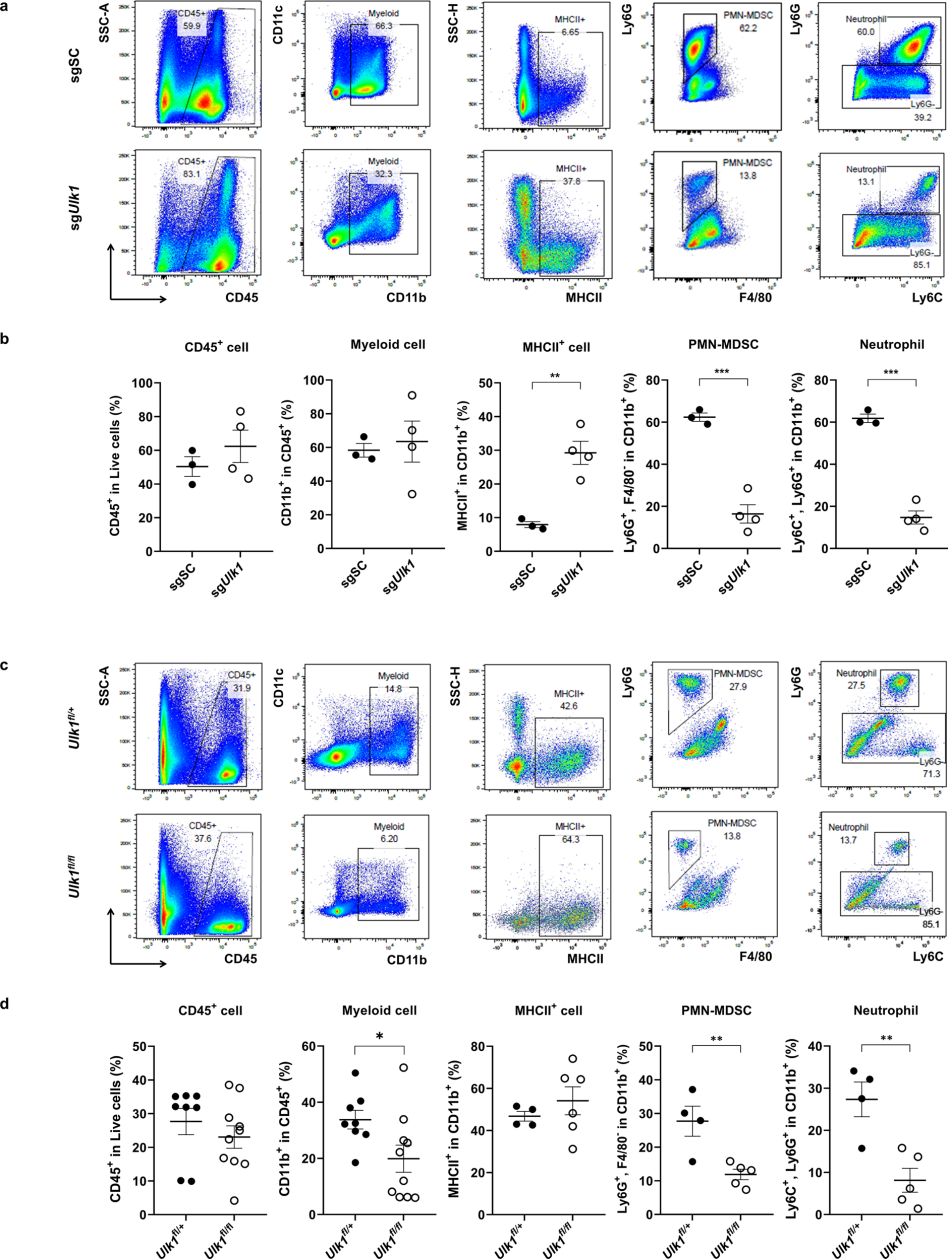

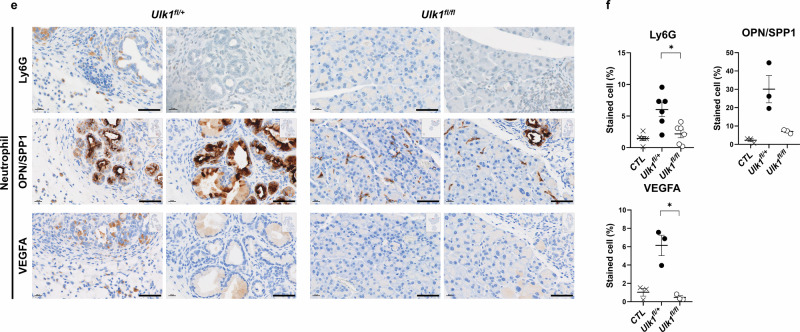
Tumor Ulk1 deletion modulates myeloid cell recruitment in both orthotopic and *KPC* GEM models. **a**,**b** Representative flow cytometry plots (**a**) and quantification (**b**) of the expression by the myeloid immune cell types analyzed by FACS in pancreas tissue from the orthotopic mouse model (*n* > 3; CD45^+^ *P* = 0.376302, myeloid cell *P* = 0.742007, MHC-II^+^ *P* = 0.003583, PMN-MDSC *P* = 0.000378 and neutrophil *P* = 0.000079). The 7-week-old female C57BL/6 mice were injected with KPC sgSC or KPC sg*Ulk1* cells and then killed 3 weeks later. **c**,**d** Representative flow cytometry plots (**c**) and quantification (**d**) of the expression by the myeloid immune cell types analyzed by FACS in pancreas tissue from the ~12–14-week-old *KPC*;*Ulk1*^*fl*/+^ and *KPC*;*Ulk1*^*fl/fl*^ GEM mice (*n* > 4; CD45^+^ *P* = 0.378793, myeloid cell *P* = 0.038968, MHC-II^+^ *P* = 0.391091, PMN-MDSC *P* = 0.007721 and neutrophil *P* = 0.005358). Error bars indicate the mean ± s.e.m. **e**,**f** Representative images (**e**) and quantitative data (**f**) of Ly6G, OPN/SPP1 and VEGFA in pancreas tissue from *KPC*;*Ulk1*^*fl*/+^ and *KPC*;*Ulk1*^*fl/fl*^ GEM mice. Scale bars are indicated in the figures, and error bars indicate the mean ± s.e.m. for over three independent experiments (Ly6G *P* = 0.0121, OPN/SPP1 *P* = 0.1019 and VEGFA *P* = 0.0482). All values were considered statistically significant by Student’s *t*-test (^*^P < 0.05; ^**^P < 0.01; ^***^P < 0.001; ^****^P < 0.0001).

Although the overall proportions of tumor-infiltrating CD45^+^ immune cells and CD45^+^CD11b^+^ myeloid cells remained unchanged between the two groups, *Ulk1* KO-derived tumors showed a marked reduction in the proportion of neutrophils (CD45^+^CD11b^+^Ly6G^+^) and PMN-MDSCs (CD11b^+^Ly6G^+^F4/80^−^) compared with tumors derived from *Ulk1* WT KPC cells, alongside a significant increase in major histocompatibility complex (MHC)-II^+^ antigen-presenting cells (APCs) (CD45^+^CD11b^+^ MHC-II^+^) in tumors derived from *Ulk1* KO KPC cells (Fig. 6a,b).

In the lymphoid compartment, *Ulk1* KO cell-derived tumors exhibited a substantial increase in the proportion of CD8^+^ T cells compared with *Ulk1* WT tumors; however, this difference did not reach statistical significance (Supplementary Fig. 8a). CD4^+^ T cell proportions were moderately decreased in *Ulk1* KO tumors, resulting in an overall increase in the CD8^+^:CD4^+^ T cell ratio relative to *Ulk1* WT tumors. Notably, the effector memory subset among CD8^+^ T cells (CD44^+^, CD62L^−^ in CD8^+^ cells) was significantly higher portion in *Ulk1* KO tumors, despite the modest change in total CD8^+^ T cell levels. This enrichment of effector memory CD8^+^ T cells may be associated with the enhanced recruitment of MHC-II^+^ APCs observed in *Ulk1* KO tumors.

To validate these findings in a spontaneous PDAC model, we performed similar immunophenotyping in KPC control and *KPC;Ulk1*^*fl/fl*^ mice (Fig. 6c,d). Similar to the orthotopic model, we observed increased proportions of MHC-II^+^ APCs and significantly reduced neutrophils and PMN-MDSCs in *KPC;Ulk1*^*fl/fl*^ mice. IHC analysis further confirmed that immune-suppressive neutrophils and PMN-MDSC markers (Ly6G, OPN/SPP1 and VEGFA) were markedly reduced in the pancreas of *KPC*;*Ulk1*^*fl/fl*^ mice (Fig. 6e,f). Notably, well-established protumorigenic neutrophil markers, OPN/SPP1 and VEGFA were significantly lower in *KPC;Ulk1*^*fl/fl*^ tumors compared with *KPC;Ulk1*^*fl*/+^tumors, consistent with the reduced Ly6G^+^ cell infiltration observed in *KPC;Ulk1*^*fl/fl*^. These results suggest that protumorigenic neutrophil subsets are more abundant in *KPC;Ulk1*^*fl*/+^tumors than in *KPC;Ulk1*^*fl/fl*^ tumors.

Within lymphoid populations, *KPC;Ulk1*^*fl/fl*^ mice showed a modest increase in CD8^+^ T cell proportions, whereas the CD4^+^ T cell proportion remained comparable to those in *KPC;Ulk1*^*fl*/+^ control mice, which was less pronounced than that observed in the syngeneic orthotopic model (Supplementary Fig. 8b).

Collectively, these data from two independent in vivo models indicate that tissue-specific *Ulk1* depletion enhances antitumor immune responses by markedly reducing immunosuppressive neutrophils and PMN-MDSCs, promoting APCs and substantially increasing CD8^+^ T cell infiltration, thereby contributing to tumor regression.

### Ulk1 regulates cytokine and chemokine secretion to support a protumorigenic immune microenvironment

To further dissect how ULK1 shapes the immune landscape in pancreatic tumors, we profiled cytokine and chemokine expression in pancreatic tissues from KPC control (*KPC*;*Ulk1*^*fl*/+^) and KPC *Ulk1* KO (*KPC*;*Ulk1*^*fl/fl*^) mice. Several protumorigenic cytokines and chemokines including ICAM-1/CD54, TIMP1, interleukin (IL)-1RN (IL-1ra) and IL-16^34,39–41^ were upregulated in KPC control pancreatic tissues compared with those in *KPC*;*Ulk1*^*fl/fl*^ mice (Supplementary Fig. 9). While Ccl2 was modestly downregulated in *KPC;Ulk1*^*fl/fl*^ tissues, Cxcl12 levels remained unchanged.

**Fig. 7:**
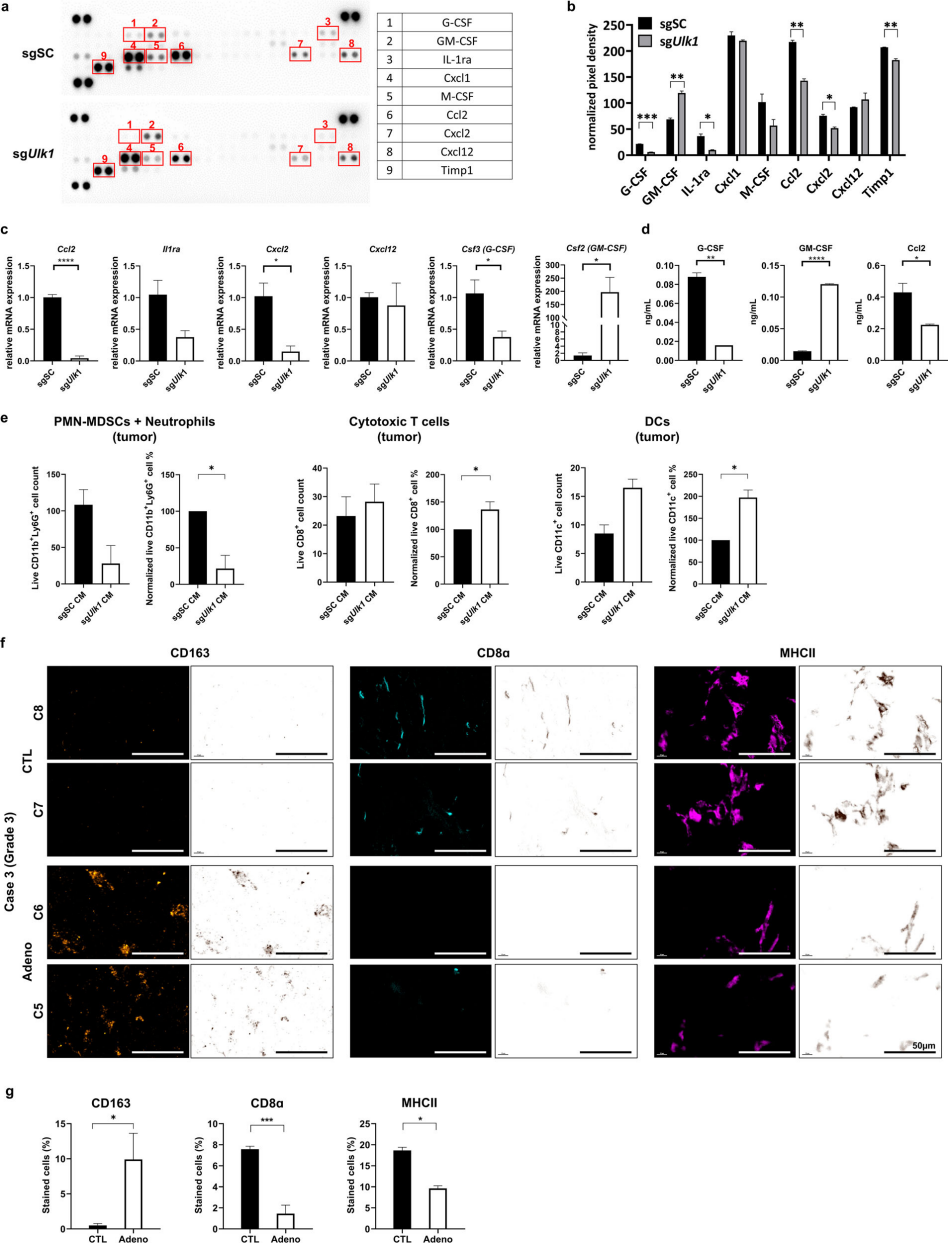
Ulk1 regulates the expression and secretion of cytokines and chemokines critical to the TME. **a**,**b** Cytokine array images (**a**) and quantification (**b**) of CM from sgSC or sg*Ulk1* KPC cells. Key differences were observed in G-CSF (*P* = 0.0002), GM-CSF (*P* = 0.0040), IL-1ra (*P* = 0.0048), Ccl2 (*P* = 0.0022), Cxcl2 (*P* = 0.0104) and Timp1 (*P* = 0.0064). Red squares indicate cytokines with notable differences between two groups, and bar graphs show pixel densities analyzed by ImageJ. **c** qPCR analysis of cytokine and chemokine mRNA in sgSC or sg*Ulk1* KPC cells. (*Ccl2* *P* < 0.0001, *Il1ra* *P* = 0.539, *Cxcl1* *P* = 0.0142, *Cxcl2* *P* = 0.0201, *Cxcl12* *P* = 0.7400 and *Csf3* *P* = 0.0258). **d** The secreted levels of cytokines from sgSC or sg*Ulk1* KPC cells using ELISA analysis (G-CSF *P* = 0.0017, GM-CSF *P* < 0.0001 and Ccl2 *P* = 0.0362). Each type of cell was seeded, incubated for 24 h and then harvested for either RNA extraction of cells or CM. Error bars indicate the mean ± s.e.m. for over three independent experiments. **e** Viability analysis of sorted immune cells cultured with CM from sgSC or sg*Ulk1* cells. Neutrophils including PMN-MDSCs (CD45^+^CD11b^+^Ly6G^+^ cells), cytotoxic T cells (CD45^+^CD8^+^ cells) and DCs (CD45^+^CD11c^+^ cells) from the pancreas in ~15–16-week-old *KPC;Ulk1*^+/*fl*^ mice were sorted and then cultured with CM from KPC sgSC or KPC sg*Ulk1* (sgSC CM or sg*Ulk1* CM) for 24 h, which were measured calcein-stained live cells using Operetta CLS. Scale bars are indicated in the figures, and error bars indicate the mean ± s.e.m. for over two independent wells (PMN-MDSCs + neutrophils in pancreas count *P* = 0.0668, normalized *P* = 0.0124; cytotoxic T cells in pancreas count *P* = 0.6024, normalized *P* = 0.3923; DCs in pancreas count *P* = 0.0637, normalized *P* = 0.0298). **f**,**g** Representative images (**f**) and quantitative data (**g**) of CD163 (orange), CD8α (cyan) and MHC-II (magenta) by multi-IHC in grade 3 human PAAD TMA. C7 and C8 cores represent cancer-adjacent pancreas tissue or adjacent normal pancreas tissue (CTL), but C5 and C6 represent adenocarcinoma (Adeno). Scale bars are indicated in the figures, and error bars indicate the mean ± s.e.m. for two independent cores (CD163 *P* = 0.0453, CD8α *P* = 0.0004 and MHC-II *P* = 0.0115). All values were considered statistically significant by Student’s *t*-test (^*^P < 0.05; ^**^P < 0.01; ^***^P < 0.001; ^****^P < 0.0001).

Cytokine and chemokine profiling of CM from *Ulk1* WT and *Ulk1* KO KPC cells revealed distinct secretory profiles, reinforcing the tumor-promoting immune landscapes (Fig. 7a,b). The most dominantly altered cytokines and chemokines between the two groups are shown in Fig. 7a,b. Icam-1/CD54 and Timp1 levels, which showed difference in pancreas tissues from GEM mice, were either undetectable or comparable in CM from KPC sgSC and KPC sg*Ulk1* cells, suggesting that these factors probably originate from stromal fibroblasts rather than from tumor epithelial cells. By contrast, protumorigenic chemokines Cxcl2 and Ccl2 —known to promote the recruitment of PMN-MDSCs and M2 macrophages^42^—were markedly reduced in *Ulk1* KO cells, consistent with previous immunophenotyping data from pancreatic tissues of spontaneous cancer model (Fig. 7). Moreover, the secreted levels of G-CSF and Ccl2 were notably reduced, but GM-CSF levels were significantly elevated in in CM from *Ulk1* KO cells, indicating that Ulk1 determines to regulate tumor-intrinsic G-CSF, Ccl2 and GM-CSF secretion oppositely (Fig. 7a,b). These findings were confirmed by quantitative PCR (qPCR), which reduced expression of *Ccl2*, *IL-1rn*, *Cxcl2* and *Csf3* (G-CSF) in *Ulk1* KO cells but increased *Csf2* (GM-CSF) expression (Fig. 7c). Enzyme-linked immunosorbent assay (ELISA) results also exhibited the concentration of Ccl2, G-CSF and GM-CSF secreted from both genotyped cells, which were consistent with their gene expression levels (Fig. 7d).

Together, these data demonstrate that *Ulk1* depletion suppresses the secretion of key cytokines and chemokines that support the recruitment of tumor-promoting immune cell subsets. However, *Ulk1* loss oppositely increased a crucial cytokine such as GM-CSF that promotes the tumor-suppressive immune cell populations, through tumor-intrinsic signaling, ultimately leading to a less immune-suppressive TME and further reduced tumor progression.

### ULK1-dependent cytokine signaling influences immune cell survival

To investigate whether tumor-derived factors directly influence tumor-infiltrated immune cell survival, we isolated freshly immune cells from either pancreas tumor tissues or the blood or spleen of tumor-bearing KPC mice at 15 weeks old and sorted neutrophils including PMN-MDSC (CD45^+^CD11b^+^Ly6G^+^), cytotoxic T cells (CD45^+^CD3^+^CD8^+^) and DCs (CD45^+^ CD11c^+^), respectively. These sorted immune cells were cultured in CM from either *Ulk1* WT or KO KPC cells for 24 h, and we then assessed their viability using calcein-AM staining. Neutrophils including PMN-MDSCs from tumors showed significantly higher viability cultured in CM from KPC WT cells (Fig. 7e and Supplementary Fig. 10a). By contrast, the viability of CD8^+^ T cells and DCs from tumors was relatively higher in *Ulk1* KO CM than in KPC WT. Interestingly, these viability differences were not observed in immune cells isolated from the blood or spleen of tumor-bearing KPC mice when cultured in the same CM, suggesting that Ulk1-dependent tumor-secreted factors specifically affect immune cells within the tumor context (Supplementary Fig. 10b,c).

Collectively, these results reveal that ULK1 orchestrates a tumor-intrinsic cytokine and chemokine program that shapes an immunosuppressive microenvironment by supporting the survival of neutrophils and PMN-MDSCs and suppressing DC and cytotoxic T cell function—mechanisms that are disrupted upon ULK1 deletion.

Finally, to evaluate the translational relevance of these findings, we analyzed TMAs from human PDAC samples. Grade 3 adenocarcinoma regions displayed higher CD163^+^ M2 macrophage staining, while the infiltration of CD8^+^ cytotoxic T cells and MHC-II^+^ APCs was lower in tumor lesions (Adeno) than in adjacent normal tissue (CTL) (Fig. 7f,g). This staining pattern was also observed in TMA of grade 1 and 2 (Supplementary Fig. 11a,b), consistent with high ULK1 activity observed in human pancreatic cancers (Fig. 1a and Supplementary Fig. 2a).

Taken together, these findings indicate that *Ulk1* deletion remodels the tumor immune microenvironment by reducing tumor-promoting myeloid cells such as M2 macrophages and neutrophils or PMN-MDSCs, and increasing cytotoxic T cells and APCs, leading to enhanced antitumor immunity and suppressed tumor growth.

## Discussion

Autophagy plays a context-dependent role in cancer, functioning as a tumor suppressor during early tumorigenesis but promoting tumor survival in established malignancies. In PDAC, elevated autophagy activity supports tumor progression by providing essential metabolic substrates for cancer cell survival. Although the importance of autophagy in PDAC has been demonstrated by the tumor-suppressive effects of Atg5 and Atg7 deletion^7,8^, the role of ULK1, a key initiator of autophagy, remains underexplored in in this context. Here, we suggest ULK1 as a critical regulator of pancreatic tumor progression through both cell-intrinsic and immune-modulatory mechanisms. Our findings support a role for ULK1 in generating a protumorigenic immune microenvironment and suggest that targeting ULK1 may offer a dual therapeutic benefit by impairing autophagy and promoting antitumor immunity.

Although *ULK1* mRNA expression is comparable between healthy patients and those with tumors from human PDAC datasets (TCGA) (Supplementary Fig. 1), our multi-IHC and immunoblot analysis revealed elevated ULK1 activity—as marked by phospho-Atg14 levels (pAtg14)—in high-grade human PDAC tissues and cells (Fig. 1), suggesting that ULK1 activity, rather than its expression, is important for tumor progression.

The functional significance of ULK1 in tumor progression is further supported by our findings that ULK1 depletion in both mouse (KPC) and human (MIA PaCa-2) pancreatic cancer cells significantly impaired their proliferation and invasion, supporting its role in tumor aggressiveness (Fig. 2 and Supplementary Fig. 3). Consistent with its crucial role in autophagy activation, ULK1-depleted cells showed defective autophagosome formation, as indicated by reduced LC3-II/I ratios, LC3-II levels and GFP-LC3 puncta under stress conditions, confirming that ULK1 is critical for maintaining autophagy flux and metabolic adaptation for cancer growth.

Importantly, our in vivo studies using both syngeneic orthotopic allografts and spontaneous KPC GEM models provide compelling evidence that Ulk1 is required for pancreatic tumor development in a physiological setting. Tumor-intrinsic deletion of *Ulk1* significantly delayed tumor onset, reduced PDAC burden and extended survival (Figs. 2–4). This study provides direct evidence to demonstrate a tumor-promoting function of Ulk1 in a spontaneous PDAC model, suggesting its potential as a promising therapeutic target.

Beyond its canonical role in autophagy-dependent tumor survival, we found an immune-modulatory function of ULK1 in the PDAC TME. Analysis of TCGA datasets revealed a negative correlation between ULK1 expression and MHC class II-mediated antigen presentation pathways (Supplementary Table 5), implicating that ULK1 may suppress antitumor immunity by inhibiting antigen processing in tumors and suppressing APCs^34,37^. While autophagy has been implicated in immune evasion, particularly through MHC-I degradation of tumor cells^37^, our data further revealed that *Ulk1*-deficient tumors markedly increased the infiltration of MHC-II^+^ APCs into tumors, potentially enhancing tumor antigen presentation and T cell priming (Fig. 5 and Supplementary Figs. 5, 7 and 8).

Moreover, genetic deletion of *Ulk1* in tumors significantly altered the composition of the TME across both orthotopic and spontaneous KPC models. We observed a marked reduction in immunosuppressive PMN-MDSCs and neutrophils. This decrease aligns with the IHC staining showing that intratumoral neutrophils enriched for SPP1 and VEGFA—markers of terminally differentiated, pro-angiogenic neutrophils—were abundant in KPC tumors but markedly reduced in *KPC;Ulk1*^*fl/fl*^ tumors, consistent with their established role in promoting tumor vascularization and growth^43,44^. Such neutrophil populations are known to adapt to metabolic constraints in the TME, including hypoxia and glycolytic reprogramming, features we observed more prominently in KPC tumors relative to their Ulk1-deficient counterparts.

Interestingly, although MHC-II^+^ APCs were significantly increased in Ulk1 depletion, CD8^+^ cytotoxic T cells showed only a substantial and not a statistically significant increase, with a notable expansion of the effector memory subset in Ulk1 KO orthotopic tumors (Figs. 5 and 6 and Supplementary Figs. 5, 7 and 8). These consistent observations across models strongly suggest that ULK1 contributes to immune evasion by orchestrating the recruitment and maintenance of suppressive myeloid populations while restricting the expansion of cytotoxic immune cells. Given that DCs were markedly increased in *Ulk1* KO tumors, we assume that effective DC-mediated T cell priming may also require additional factors and distinct TME niches.

Mechanistically, cytokine and chemokine profiling revealed that Ulk1-deficient tumors secreted lower levels of Ccl2, Cxcl2 and G-CSF, key factors that recruit immunosuppressive myeloid cells^45–54^. Interestingly, among the cytokines, GM-CSF was the most prominently elevated in *Ulk1* KO tumors (Fig. 7). Although there are reports of dual roles of GM-CSF in the TME by promoting or inhibiting immune cell subtypes^55–57^, recently, GM-CSF shows antitumor immune responses by activating M1 macrophages and enhancing DC differentiation^58–60^. The upregulation of DC populations may explain the increased viability of APCs and may have further activated T cells observed in *Ulk1*-deficient tumors (Figs. 5 and 6 and Supplementary Figs. 5 and 6). These tumor-intrinsic cytokine changes were recapitulated in vitro using CM from *Ulk1* KO KPC cells, confirming that *Ulk1*-mediated tumor signaling influences immune cell recruitment as determined by the types of cytokine and chemokine (Fig. 7a–d).

Functional assays further demonstrated that CM from *Ulk1* KO cells impaired the survival of tumor-infiltrating neutrophils including PMN-MDSCs, while enhancing the viability of DCs and CD8^+^ T cells (Fig. 7e–g and Supplementary Fig. 10). These results suggest that Ulk1-dependent cytokine secretion dictates immune cell fate within the tumor niche. Overall, these findings support the role of Ulk1 in maintaining an immunosuppressive TME, promoting tumor growth not only through autophagy-driven metabolic support but also by modulating the balance of immune cell compositions and dynamics, the latter of which requires further mechanistic investigation under physiological TME conditions.

Previous studies have shown that autophagy promotes the secretion of protumorigenic factors—including IL-6, IL-8 and MMP2—in Ras-driven cancers and facilitates IL-1β secretion through unconventional secretory pathways^61^. In line with these studies, our data indicate that ULK1 plays an important role in regulating the secretion of cytokine and chemokines that reprogram the tumor immune landscape. While the precise molecular mechanisms by which ULK1 regulates cytokine expression and trafficking remain to be fully elucidated, our findings strongly implicate ULK1-mediated tumor signals as playing a crucial role in coordinating tumor–immune interactions through selective modulation of immune-regulatory chemokines and cytokines.

A growing number of evidence suggests that ULK1 exerts autophagy-independent functions that also contribute to tumor progression. Beyond its canonical role in autophagy initiation, ULK1 regulates various cancer-related processes—including metabolic reprogramming, redox homeostasis, cell cycle progression and immune modulation—through direct phosphorylation of diverse downstream targets^62,63^. For instance, ULK1/2 phosphorylates key glycolytic enzymes such as hexokinase 1, phosphofructokinase 1 and enolase 1, thereby diverting glucose flux toward the pentose phosphate pathway to maintain redox balance under metabolic stress, independent of autophagy^64^. More recently, ULK1 was shown to directly phosphorylate lactate dehydrogenase A, enhancing lactate production and promoting Vps34 lactylation, which is associated with cell homeostasis and growth mediated by noncanonical autophagy induction^65^. These findings highlight that ULK1 acts as a key integrator of metabolic reprogramming and autophagic signaling.

ULK1 also contributes to the cell cycle to facilitate cell proliferation. It phosphorylates the spindle assembly checkpoint protein Mad1, facilitating its recruitment to kinetochores during mitosis and ensuring chromosome segregation fidelity^66^. Furthermore, phosphorylation of ULK1 and its binding protein by cyclin-dependent kinase 1/cyclin B promotes mitotic entry and cell cycle progression, as indicated by impaired cancer growth upon genetic deletion of ULK1 or ATG13^67^. As evidence of cell death regulation, ULK1 directly phosphorylates RIPK1, thereby inhibiting the formation of the necrosome complex and protecting cells from tumor necrosis factor-induced necroptosis^68^.

In terms of innate immune regulation, ULK1 shows context-dependent, autophagy-independent roles. ULK1 negatively regulates antiviral responses by phosphorylating STING and dampening type I interferon signaling^69^. Conversely, ULK1 enhances antiviral immunity by promoting type I interferon-stimulated gene expression via activation of p38 MAP kinase^70^. These apparently opposing roles imply the importance of ULK1 in balancing antiviral defense with protection against chronic inflammation.

Collectively, these findings indicate the multifaceted, autophagy-independent roles of ULK1 in cancer progression. Taken together with our findings—that ULK1 promotes pancreatic cancer progression by supporting tumor metabolism and orchestrating an immunosuppressive TME through cytokine-mediated immune cell modulation—we suggest that ULK1 serves as a key regulator of both tumor-intrinsic and immune-regulatory pathways, even independent of its role in autophagy. Therefore, targeting ULK1 may offer a dual therapeutic strategy in PDAC by disrupting metabolic support and enhancing antitumor immunity.

## Supplementary information

Supplementary Information

## Data Availability

For gene set enrichment analysis, the TCGA database was accessed in the Cancer Integrative Platform (cBioportal; http://www.cBioportal.mskcc.org/). For the survival analysis, the latest TCGA iteration of transcriptome and clinical data was downloaded directly using the application programming interface provided by TCGAbiolinks (version 2.30.4) in R (4.3.1). All other datasets supporting the current study are available from the corresponding author upon reasonable request.

## References

[1] He, C. & Klionsky, D. J. Regulation mechanisms and signaling pathways of autophagy. *Annu. Rev. Genet.* **43**, 67–93 (2009). 19653858 10.1146/annurev-genet-102808-114910 PMC2831538

[2] Levine, B. & Kroemer, G. Biological functions of autophagy genes: a disease perspective. *Cell* **176**, 11–42 (2019). 30633901 10.1016/j.cell.2018.09.048 PMC6347410

[3] Guo, J. Y. et al. Activated Ras requires autophagy to maintain oxidative metabolism and tumorigenesis. *Genes Dev.* **25**, 460–470 (2011). 21317241 10.1101/gad.2016311 PMC3049287

[4] Guo, J. Y. et al. Autophagy suppresses progression of K-ras-induced lung tumors to oncocytomas and maintains lipid homeostasis. *Genes Dev.* **27**, 1447–1461 (2013). 23824538 10.1101/gad.219642.113 PMC3713426

[5] Rao, S. et al. A dual role for autophagy in a murine model of lung cancer. *Nat. Commun.* **5**, 3056 (2014). 24445999 10.1038/ncomms4056

[6] Xie, X., Koh, J. Y., Price, S., White, E. & Mehnert, J. M. Atg7 overcomes senescence and promotes growth of BrafV600E-driven melanoma. *Cancer Discov.* **5**, 410–423 (2015). 25673642 10.1158/2159-8290.CD-14-1473 PMC4390491

[7] Yang, A. et al. Autophagy is critical for pancreatic tumor growth and progression in tumors with p53 alterations. *Cancer Discov.* **4**, 905–913 (2014). 24875860 10.1158/2159-8290.CD-14-0362 PMC4125497

[8] Yang, S. et al. Pancreatic cancers require autophagy for tumor growth. *Genes Dev.* **25**, 717–729 (2011). 21406549 10.1101/gad.2016111 PMC3070934

[9] Wei, H. et al. Suppression of autophagy by FIP200 deletion inhibits mammary tumorigenesis. *Genes Dev.* **25**, 1510–1527 (2011). 21764854 10.1101/gad.2051011 PMC3143941

[10] Taraborrelli, L. et al. Tumor-intrinsic expression of the autophagy gene Atg16l1 suppresses anti-tumor immunity in colorectal cancer. *Nat. Commun.* **14**, 5945 (2023). 37741832 10.1038/s41467-023-41618-7 PMC10517947

[11] Yang, A. et al. Autophagy sustains pancreatic cancer growth through both cell-autonomous and nonautonomous mechanisms. *Cancer Discov.* **8**, 276–287 (2018). 29317452 10.1158/2159-8290.CD-17-0952 PMC5835190

[12] Papinski, D. & Kraft, C. Regulation of autophagy by signaling through the Atg1/ULK1 complex. *J. Mol. Biol.* **428**, 1725–1741 (2016). 27059781 10.1016/j.jmb.2016.03.030

[13] Hosokawa, N. et al. Nutrient-dependent mTORC1 association with the ULK1-Atg13-FIP200 complex required for autophagy. *Mol. Biol. Cell* **20**, 1981–1991 (2009). 19211835 10.1091/mbc.E08-12-1248 PMC2663915

[14] Jung, C. H. et al. ULK-Atg13-FIP200 complexes mediate mTOR signaling to the autophagy machinery. *Mol. Biol. Cell* **20**, 1992–2003 (2009). 19225151 10.1091/mbc.E08-12-1249 PMC2663920

[15] Ganley, I. G. et al. ULK1.ATG13.FIP200 complex mediates mTOR signaling and is essential for autophagy. *J. Biol. Chem.* **284**, 12297–12305 (2009). 19258318 10.1074/jbc.M900573200 PMC2673298

[16] Russell, R. C. et al. ULK1 induces autophagy by phosphorylating Beclin-1 and activating VPS34 lipid kinase. *Nat. Cell Biol.* **15**, 741–750 (2013). 23685627 10.1038/ncb2757 PMC3885611

[17] Park, J. M. et al. The ULK1 complex mediates MTORC1 signaling to the autophagy initiation machinery via binding and phosphorylating ATG14. *Autophagy* **12**, 547–564 (2016). 27046250 10.1080/15548627.2016.1140293 PMC4835982

[18] Wei, Y. et al. The stress-responsive kinases MAPKAPK2/MAPKAPK3 activate starvation-induced autophagy through Beclin 1 phosphorylation. *eLife* **4** e05289 (2015). 10.7554/eLife.05289 PMC4337728 25693418

[19] Kim, J., Kundu, M., Viollet, B. & Guan, K. L. AMPK and mTOR regulate autophagy through direct phosphorylation of Ulk1. *Nat. Cell Biol.* **13**, 132–141 (2011). 21258367 10.1038/ncb2152 PMC3987946

[20] Zachari, M. & Ganley, I. G. The mammalian ULK1 complex and autophagy initiation. *Essays Biochem.* **61**, 585–596 (2017). 29233870 10.1042/EBC20170021 PMC5869855

[21] Cheong, H., Lindsten, T., Wu, J., Lu, C. & Thompson, C. B. Ammonia-induced autophagy is independent of ULK1/ULK2 kinases. *Proc. Natl Acad. Sci. USA* **108**, 11121–11126 (2011). 21690395 10.1073/pnas.1107969108 PMC3131371

[22] Cheong, H. et al. Analysis of a lung defect in autophagy-deficient mouse strains. *Autophagy* **10**, 45–56 (2014). 24275123 10.4161/auto.26505 PMC4028323

[23] Mizushima, N. & Levine, B. Autophagy in mammalian development and differentiation. *Nat. Cell Biol.* **12**, 823–830 (2010). 20811354 10.1038/ncb0910-823 PMC3127249

[24] Kundu, M. et al. Ulk1 plays a critical role in the autophagic clearance of mitochondria and ribosomes during reticulocyte maturation. *Blood* **112**, 1493–1502 (2008). 18539900 10.1182/blood-2008-02-137398 PMC2515143

[25] Amaravadi, R. K., Kimmelman, A. C. & Debnath, J. Targeting autophagy in cancer: recent advances and future directions. *Cancer Discov.* **9**, 1167–1181 (2019). 31434711 10.1158/2159-8290.CD-19-0292 PMC7306856

[26] Lee, C. S. et al. MAP kinase and autophagy pathways cooperate to maintain RAS mutant cancer cell survival. *Proc. Natl Acad. Sci. USA* **116**, 4508–4517 (2019). 30709910 10.1073/pnas.1817494116 PMC6410784

[27] Kinsey, C. G. et al. Protective autophagy elicited by RAF→MEK→ERK inhibition suggests a treatment strategy for RAS-driven cancers. *Nat. Med.* **25**, 620–627 (2019). 30833748 10.1038/s41591-019-0367-9 PMC6452642

[28] Bryant, K. L. et al. Combination of ERK and autophagy inhibition as a treatment approach for pancreatic cancer. *Nat. Med.* **25**, 628–640 (2019). 30833752 10.1038/s41591-019-0368-8 PMC6484853

[29] Egan, D. F. et al. Small molecule inhibition of the autophagy kinase ULK1 and identification of ULK1 substrates. *Mol. Cell* **59**, 285–297 (2015). 26118643 10.1016/j.molcel.2015.05.031 PMC4530630

[30] Ren, H. et al. Design, synthesis, and characterization of an orally active dual-specific ULK1/2 autophagy inhibitor that synergizes with the PARP inhibitor olaparib for the treatment of triple-negative breast cancer. *J. Med Chem.* **63**, 14609–14625 (2020). 33200929 10.1021/acs.jmedchem.0c00873 PMC8064294

[31] Ghazi, P. C. et al. Inhibition of ULK1/2 and KRAS(G12C) controls tumor growth in preclinical models of lung cancer. *eLife* **13**, RP96992 (2024). 10.7554/eLife.96992 PMC11364435 39213022

[32] Sanjana, N. E., Shalem, O. & Zhang, F. Improved vectors and genome-wide libraries for CRISPR screening. *Nat. Methods* **11**, 783–784 (2014). 25075903 10.1038/nmeth.3047 PMC4486245

[33] Hingorani, S. R. et al. Trp53R172H and KrasG12D cooperate to promote chromosomal instability and widely metastatic pancreatic ductal adenocarcinoma in mice. *Cancer Cell* **7**, 469–483 (2005). 15894267 10.1016/j.ccr.2005.04.023

[34] Liou, G. Y. et al. Mutant KRAS-induced expression of ICAM-1 in pancreatic acinar cells causes attraction of macrophages to expedite the formation of precancerous lesions. *Cancer Discov.* **5**, 52–63 (2015). 25361845 10.1158/2159-8290.CD-14-0474 PMC4293204

[35] Krug, K. et al. A curated resource for phosphosite-specific signature analysis. *Mol. Cell Proteom.* **18**, 576–593 (2019). 10.1074/mcp.TIR118.000943 PMC6398202 30563849

[36] Nofech-Mozes, I., Soave, D., Awadalla, P. & Abelson, S. Pan-cancer classification of single cells in the tumour microenvironment. *Nat. Commun.* **14**, 1615 (2023). 36959212 10.1038/s41467-023-37353-8 PMC10036554

[37] Yamamoto, K. et al. Autophagy promotes immune evasion of pancreatic cancer by degrading MHC-I. *Nature* **581**, 100–105 (2020). 32376951 10.1038/s41586-020-2229-5 PMC7296553

[38] Lawson, K. A. et al. Functional genomic landscape of cancer-intrinsic evasion of killing by T cells. *Nature* **586**, 120–126 (2020). 32968282 10.1038/s41586-020-2746-2 PMC9014559

[39] Justo, B. L. & Jasiulionis, M. G. Characteristics of TIMP1, CD63, and beta1-integrin and the functional impact of their interaction in cancer. *Int. J. Mol. Sci*. **22**, 9319 (2021). 10.3390/ijms22179319 PMC8431149 34502227

[40] Fan, Y. C. et al. Tumor-derived interleukin-1 receptor antagonist exhibits immunosuppressive functions and promotes pancreatic cancer. *Cell Biosci.* **13**, 147 (2023). 37563620 10.1186/s13578-023-01090-8 PMC10416534

[41] Yang, S. J. et al. Neutralizing IL-16 enhances the efficacy of targeting Aurora-A therapy in colorectal cancer with high lymphocyte infiltration through restoring anti-tumor immunity. *Cell Death Dis.* **15**, 103 (2024). 38291041 10.1038/s41419-023-06381-z PMC10828506

[42] Kohli, K., Pillarisetty, V. G. & Kim, T. S. Key chemokines direct migration of immune cells in solid tumors. *Cancer Gene Ther.* **29**, 10–21 (2022). 33603130 10.1038/s41417-021-00303-x PMC8761573

[43] Ng, M. S. F. et al. Deterministic reprogramming of neutrophils within tumors. *Science* **383**, eadf6493 (2024). 38207030 10.1126/science.adf6493 PMC11087151

[44] Wu, Y. et al. Neutrophil profiling illuminates anti-tumor antigen-presenting potency. *Cell* **187**, 1422–1439 (2024). 38447573 10.1016/j.cell.2024.02.005

[45] Mempel, T. R., Lill, J. K. & Altenburger, L. M. How chemokines organize the tumour microenvironment. *Nat. Rev. Cancer* **24**, 28–50 (2024). 38066335 10.1038/s41568-023-00635-w PMC11480775

[46] Chun, E. et al. CCL2 promotes colorectal carcinogenesis by enhancing polymorphonuclear myeloid-derived suppressor cell population and function. *Cell Rep.* **12**, 244–257 (2015). 26146082 10.1016/j.celrep.2015.06.024 PMC4620029

[47] Yang, H. et al. CCL2–CCR2 axis recruits tumor associated macrophages to induce immune evasion through PD-1 signaling in esophageal carcinogenesis. *Mol. Cancer* **19**, 41 (2020). 32103760 10.1186/s12943-020-01165-x PMC7045401

[48] Chang, A. L. et al. CCL2 produced by the glioma microenvironment is essential for the recruitment of regulatory T cells and myeloid-derived suppressor cells. *Cancer Res.* **76**, 5671–5682 (2016). 27530322 10.1158/0008-5472.CAN-16-0144 PMC5050119

[49] Martin, T. D. et al. The adaptive immune system is a major driver of selection for tumor suppressor gene inactivation. *Science* **373**, 1327–1335 (2021). 34529489 10.1126/science.abg5784 PMC13397623

[50] Chao, T., Furth, E. E. & Vonderheide, R. H. CXCR2-dependent accumulation of tumor-associated neutrophils regulates T-cell immunity in pancreatic ductal adenocarcinoma. *Cancer Immunol. Res.* **4**, 968–982 (2016). 27737879 10.1158/2326-6066.CIR-16-0188 PMC5110270

[51] Jablonska, J., Wu, C. F., Andzinski, L., Leschner, S. & Weiss, S. CXCR2-mediated tumor-associated neutrophil recruitment is regulated by IFN-beta. *Int. J. Cancer* **134**, 1346–1358 (2014). 24154944 10.1002/ijc.28551

[52] Zhang, R. et al. PMN-MDSCs modulated by CCL20 from cancer cells promoted breast cancer cell stemness through CXCL2–CXCR2 pathway. *Signal Transduct. Target Ther.* **8**, 97 (2023). 36859354 10.1038/s41392-023-01337-3 PMC9977784

[53] Waight, J. D., Hu, Q., Miller, A., Liu, S. & Abrams, S. I. Tumor-derived G-CSF facilitates neoplastic growth through a granulocytic myeloid-derived suppressor cell-dependent mechanism. *PLoS ONE* **6**, e27690 (2011). 22110722 10.1371/journal.pone.0027690 PMC3218014

[54] Fridlender, Z. G. et al. Polarization of tumor-associated neutrophil phenotype by TGF-beta: ‘N1’ versus ‘N2’ TAN. *Cancer Cell* **16**, 183–194 (2009). 19732719 10.1016/j.ccr.2009.06.017 PMC2754404

[55] Kumar, A., Taghi Khani, A., Sanchez Ortiz, A. & Swaminathan, S. GM-CSF: a double-edged sword in cancer immunotherapy. *Front. Immunol.* **13**, 901277 (2022). 35865534 10.3389/fimmu.2022.901277 PMC9294178

[56] Bayne, L. J. et al. Tumor-derived granulocyte-macrophage colony-stimulating factor regulates myeloid inflammation and T cell immunity in pancreatic cancer. *Cancer Cell* **21**, 822–835 (2012). 22698406 10.1016/j.ccr.2012.04.025 PMC3575028

[57] Nebiker, C. A. et al. GM-CSF production by tumor cells is associated with improved survival in colorectal cancer. *Clin. Cancer Res.* **20**, 3094–3106 (2014). 24737547 10.1158/1078-0432.CCR-13-2774

[58] Greter, M. et al. GM-CSF controls nonlymphoid tissue dendritic cell homeostasis but is dispensable for the differentiation of inflammatory dendritic cells. *Immunity* **36**, 1031–1046 (2012). 22749353 10.1016/j.immuni.2012.03.027 PMC3498051

[59] Urdinguio, R. G. et al. Immune-dependent and independent antitumor activity of GM-CSF aberrantly expressed by mouse and human colorectal tumors. *Cancer Res.* **73**, 395–405 (2013). 23108143 10.1158/0008-5472.CAN-12-0806

[60] Van Overmeire, E. et al. M-CSF and GM-CSF receptor signaling differentially regulate monocyte maturation and macrophage polarization in the tumor microenvironment. *Cancer Res.* **76**, 35–42 (2016). 26573801 10.1158/0008-5472.CAN-15-0869

[61] Lock, R., Kenific, C. M., Leidal, A. M., Salas, E. & Debnath, J. Autophagy-dependent production of secreted factors facilitates oncogenic RAS-driven invasion. *Cancer Discov.* **4**, 466–479 (2014). 24513958 10.1158/2159-8290.CD-13-0841 PMC3980002

[62] Zou, L. et al. Autophagy and beyond: unraveling the complexity of UNC-51-like kinase 1 (ULK1) from biological functions to therapeutic implications. *Acta Pharm. Sin. B* **12**, 3743–3782 (2022). 36213540 10.1016/j.apsb.2022.06.004 PMC9532564

[63] Zhu, L., Li, Z., Wang, H., Cheng, Z. & Zhang, L. Unraveling the mysterious veil of ULK1: from non-canonical functions to therapeutic applications. *Int. J. Biol. Macromol.* **318**, 145177 (2025). 40505922 10.1016/j.ijbiomac.2025.145177

[64] Li, T. Y. et al. ULK1/2 constitute a bifurcate node controlling glucose metabolic fluxes in addition to autophagy. *Mol. Cell* **62**, 359–370 (2016). 27153534 10.1016/j.molcel.2016.04.009

[65] Jia, M. et al. ULK1-mediated metabolic reprogramming regulates Vps34 lipid kinase activity by its lactylation. *Sci. Adv.* **9**, eadg4993 (2023). 37267363 10.1126/sciadv.adg4993 PMC10413652

[66] Yuan, F. et al. ULK1 phosphorylates Mad1 to regulate spindle assembly checkpoint. *Nucleic Acids Res.* **47**, 8096–8110 (2019). 31291454 10.1093/nar/gkz602 PMC6736072

[67] Li, Z. et al. ULK1–ATG13 and their mitotic phospho-regulation by CDK1 connect autophagy to cell cycle. *PLoS Biol.* **18**, e3000288 (2020). 32516310 10.1371/journal.pbio.3000288 PMC7282624

[68] Wu, W. et al. The autophagy-initiating kinase ULK1 controls RIPK1-mediated cell death. *Cell Rep.* **31**, 107547 (2020). 32320653 10.1016/j.celrep.2020.107547

[69] Konno, H., Konno, K. & Barber, G. N. Cyclic dinucleotides trigger ULK1 (ATG1) phosphorylation of STING to prevent sustained innate immune signaling. *Cell* **155**, 688–698 (2013). 24119841 10.1016/j.cell.2013.09.049 PMC3881181

[70] Saleiro, D. et al. Central role of ULK1 in type I interferon signaling. *Cell Rep.* **11**, 605–617 (2015). 25892232 10.1016/j.celrep.2015.03.056 PMC4477687

