# Supplementary material for: ULK1 knockout suppresses pancreatic cancer progression by inhibiting autophagy and enhancing antitumor immunity
Source: Exp Mol Med. 2025 Dec 17;57(12):2816–32. doi: 10.1038/s12276-025-01590-2 (PMC12800254; doi:10.1038/s12276-025-01590-2)
Supplement: Supplementary file 1 — Supplementary Information [file 12276_2025_1590_MOESM1_ESM.docx]

**Supplementary Information**

**ULK1 Knockout suppresses Pancreatic Cancer Progression by Inhibiting Autophagy and Enhancing Anti-tumor Immunity**

Hana Jeong^1*^, Jinju Lee^1*^, Jiyoon Son^1*^, Jinkyung Lee^1,2^, Miju Kang^1^, Sunghyeon Cho^1,2^, Ji Hyeon Kim^1,2^, Yoon Jeon^1^, Jonghyun Lee^3^, Dongkwan Shin^3^, Hye-ran Kim^1^, Ho Lee^2^ and Heesun Cheong^1,2#^

1. Division of Cancer Biology, Research Institute National Cancer Center, Goyang 10408, Korea.

2. Department of Cancer Biomedical Science, National Cancer Center Graduate School of Cancer Science and Policy, Goyang 10408, Korea.

3. Bioinformatics Branch, Division of Cancer Data Science, Research Institute, National Cancer Center, Goyang, 10408, Korea.

**Supplementary Materials and Methods**

**Generation of stable cell lines**

GFP-LC3 was stably expressed in MIA PaCa-2 cells using a retroviral vector following standard protocols for viral transduction. For generating ULK1 knockdown cell lines, Lenti-viral vector (pLKO.1; Addgene) expressing shRNA against ULK1 was constructed. The following shRNA sequences were used for the constructs (Supplementary Table 2). Stable knockdown cells were generated using the lentiviral vector harboring either shULK1 or a scramble shRNA as a control following standard protocols for viral transduction.

For shRNA stable cell line generation, lentiviral vector for Ulk1 shRNA or control pLKO.1 vector (Sigma Cat # SHC001) was employed. For the lentivirus production, HEK-293T cells were co-transfected with the three-vector system including pLKO.1-shRNA vector and packaging vectors psPAX2 (Addgene #12260) and pMD2.G (Addgene #12259). Prior to infection, cell culture supernatant was passed through 0.45 μm syringe filter and the filtered virus was added to human pancreatic cancer cells in the presence of polybrene (10 μg/ml, Sigma Cat #TR-1003-G) and selected with Puromycin (Sigma Cat #P9620) 72 hrs post infection. Subsequently after appropriate selection steps, immunoblotting was performed to test the expression of the proper gene sets in stable cell lines. Cells were transfected with Lipofectamine 2000 (11668-019, Invitrogen) with plasmid DNAs in Opti-MEM media (31985070, Gibco) following standard protocol. GFP-LC3 in the MigRI-based retroviral vector was generously provided by Craig B. Thompson. KPC stably expressing GFP-LC3 were generated following standard protocols for retrovirus transduction. To generate sgSC (control) and sg*Ulk1* (*Ulk1* Knock-out) KPC cell line, sgRNA target sites were designed by addgene (Addgene #80263) and broad institute portal (http://portals. Broadinstitute.org/gppx/crispick/public), respectively. sgRNA target sequences are indicated in Supplementary Table 2, and lentiCRISPR v2 viral vector for each target sgRNAs was employed. For the lentivirus production, HEK-293T cells were co-transfected with the three-vector system including packaging vectors pMDLg/pRRE (Addgene #12251), pMD2.G (Addgene #12259), and pRSV-Rev (Addgene #12253). Prior to infection, cell culture supernatant was passed through 0.45 μm syringe filter and the filtered virus was added to KPC cell line in the presence of polybrene (6 μg/ml, Sigma Cat #TR-1003-G) and selected with Puromycin (Sigma Cat #P9620) 72 hrs post infection.

**Fluorescence microscopy analysis of autophagy**

Cell lines stably expressing LC3B tagged with GFP were used for monitoring autophagy activity by confocal fluorescence microscopy. Cells stably expressing GFP-LC3 construct were also used. MIA PaCa-2 stably expressing GFP-LC3 and transfected with either control or ULK1 shRNA were cultured in a glass-bottomed chamber (155409PK, Thermo Fisher Scientific Inc.) overnight, and then replaced with DMEM culture medium containing the indicated chemicals or starvation media for the indicated time periods. Nuclei were stained using Hoechst 33342. Images were acquired with the LSM780 confocal fluorescent microscope (Carl Zeiss) and the percent of either GFP-LC3 puncta area or mCherry-LC3 puncta area were normalized to the Hoechst 33342-stained area, which was quantified using ZEN black software (Carl Zeiss). The area of LC3 puncta was counted in five different arbitrary areas from three independent experiments.

**Western blotting**

Cells and tissues were harvested in ice-cold RIPA lysis buffer (50 mM Tris-Cl, pH 7.4, 150 mM NaCl, 1 % NP-40, 0.5% Na-deoxycholate, 0.1% SDS, 1 mM EDTA) containing protease inhibitor cocktail (11697498001, Roche) and phosphatase inhibitor (P5726, P0044, Sigma-Aldrich). Additionally, the tissues were grinded using TissueLyser II (QIAGEN). Soluble lysate fractions were isolated by centrifugation at 20,000× g, for 20 min at 4 ℃ and quantified using the Pierce bicinchoninic acid (BCA) Protein Assay kit (23227, Thermo Fisher Scientific Inc.). Samples were resolved by SDS polyacrylamide gel electrophoresis using equal concentrations of protein and transferred to polyvinylidene fluoride membranes (IPVH00010, Merck Millipore). The membranes were blocked with 5 % skim milk dissolved in TBS-T for 1 h and incubated overnight at 4 °C with primary antibodies. Membranes were washed with TBS-T and incubated with secondary antibodies (A90-116P, A120-101P, Bethyl Laboratories) for 1 h at room temperature. Image J software (NIH) was used for quantification of the indicated bands.

**FDG-positron emission tomography/computed tomography (PET/CT) scanning**

[F-18]FDG (2-Deoxy-2-[F-18]fluoro-D-glucose) was prepared by an automated synthesizer (The NEPTIS^®^ Nx3 system, ORA) using [F-18]fluoride generated from our on-site cyclotron (RDS-111, Siemens). All mice were fasted for 6 h before PET/CT scans supplying only water. Anesthesia was administered using 2 % isoflurane /100 % O_2_, and 18.5 MBq of [F-18]FDG was intravenously injected to each mouse. Their body temperature was controlled throughout all the imaging processes using a heating lamp. We used an eXplore Vista PET/CT system (GE healthcare), and normalization, scatter correction and attenuation correction were applied for the PET scans acquired for 5 min per bed position. The obtained images were reconstructed with iterative reconstruction (OSEM 2-D, 32 subsets, two interactions). For CT scans, the X-ray conditions were: 250 μA and 40 kV for 5 min. The CT resolution was 200 μm, and the number of acquired projections was 360. All image analysis were done using OsiriX MD software ([www.osirix-viewer.com](http://www.osirix-viewer.com), Pixmeo SARL).

**Quantitative real-time PCR**

Total cellular RNA was extracted with TRIzol (15596-018, Invitrogen) using chloroform, precipitated with isopropyl alcohol, washed with 70% ethanol, and eluted in RNase-free water. The concentration of the isolated RNA was measured using a Nanodrop 2000 spectrophotometer (Thermo Scientific Inc.) at 260 nm. The cDNAs used as templates in RT‒qPCR were prepared using 1 ug of total RNA. mRNA expression was evaluated by RT‒qPCR using AccuPower® 2x GreenStar^TM^ qPCR Master Mix (K-6254, Bioneer) and normalized to *ActB* expression in each sample. For PCR, DNA polymerase activation was performed for 5 min at 95 °C, and amplification was then conducted in a Light Cycler 480 instrument II (Roche). The primer sequences used in these experiments are listed in Supplementary Table 2.

**RNA-Sequencing analysis**

Total RNA was extracted and quality assessed using FastQC (v0.11.7). Adapter trimming and quality filtering were performed with Trimmomatic (v0.38). RNA-seq libraries were prepared using the Illumina TruSeq Stranded mRNA Library Prep Kit. Reads were aligned to the mouse reference genome (NCBI_108/mm10) using HISAT2 (v2.1.0). Transcript assembly and quantification were performed with StringTie (v2.1.3b) to generate a gene-level count matrix. The resulting raw counts were normalized using the Trimmed Mean of M-values (TMM) method via the calcNormFactors function in the edgeR package in R. Differential expression analysis was conducted using exactTest function in edgeR. DEGs were defined as those with |log2 fold change; FC| > 2 and p.value < 0.05.

For visualization, volcano plots were generated using log2 fold changes and p-values derived from comparisons between WT-com vs. KO-com (Supplementary Fig. 6B) and WT-AA vs. KO-AA (Supplementary Fig. 6C). Heatmaps were created by applying z-score scaling to the normalized expression values, followed by hierarchical clustering using the Ward.D2 method. All visualizations were performed in R version 4.3.1

**Protein extraction and peptide digestion**

hTERT-immortalized human pancreatic normal epithelial (HPNE) cells infected by KRas^G12D^ (MigRI- KRAS^G12D^) (HPNE-KRas) were used for proteomics analysis in the treatment with either vehicle or 25 μM chloroquine for 24 h. Cell pellets were solubilized in SDS solubilization buffer (5% SDS, 50mM TEAB pH 8.5) using S220 Focused-ultrasonicator (Covaris). Proteins were digested using S-Trap™ spin columns (Protifi, Huntington, NY) with manufacturer’s instructions. The samples were reduced by DTT and alkylated by iodoacetamide (IAA). After quenching the alkylation reaction, additional SDS and phosphoric acid were added so that the final concentration was 5% SDS and 1.2% phosphoric acid. Acidified samples were mixed with 90% methanol in 100mM TEAB, loaded into the S-Trap micro columns, incubated with mass spec grade trypsin/LysC (Promega) for 3 h at 47°C. Eluted peptides were evaporated using vacuum concentrator and cleaned up using C18 spin columns (Thermo Fisher Scientific).

**TMTpro Labeling**

Desalted peptide samples were reconstituted in 100mM TEAB pH 8.5, labeled using TMTpro reagents (Thermo Fisher Scientific). Each prepared TMTpro reagent was transferred to the peptide sample, the mixture was incubated for 1 h, quenched by addition of 5 mL of 5% hydroxylamine and incubated for 15 min at room temperature. Differently labeled 4plex peptides was pooled and dried using vacuum concentrator.

**Peptide fractionation by Mid-pH Reverse Phase Liquid Chromatography**

The pooled 4plex TMTpro-labeled sample was separated using Agilent 1260 Infinity HPLC system (Agilent, Palo Alto, CA). A Xbridge C18 analytical column (4.6 mm × 250 mm, 130 Å, 5 um) and a guard column (4.6 mm × 20 mm, 130 Å, 5 um) were used for peptide separation. Solvents A and B were 10 mM triethylammonium bicarbonate (TEAB) in water (pH 7.5) and 10 mM TEAB in 90% acetonitrile (ACN, pH 7.5), respectively. Peptide fractionation was performed using a 120 min gradient at a flow rate of 500 mL/min as follows: 0% solvent B for 15 min, 0 to 5% solvent B over 10 min, from 5 to 35% solvent B over 60 min, from 35 to 70% solvent B over 15 min, 70% solvent B for 10 min, from 70 to 0% solvent B over 10 min. A total of 96 fractions were collected every minute from 15 to 110 min and were pooled into 24 non-continuous peptide fractions (i.e., #1–#25–#49–#73, #2–#26–#50–#74, …, #24–#48–#72–#96) and dried using concentrator.

**LC-MS/MS analysis**

TMTpro-labeled peptides prepared for global proteome analysis were resuspended with 0.1% formic acid in water, separated using an Ultimate 3000 RSLCnano system (Thermo Scientific) and analyzed by an Orbitrap Eclipse Tribrid mass spectrometer (Thermo Scientific). Solvents A and B were 0.1% FA in water and 0.1% FA in acetonitrile, respectively. The peptides were loaded onto trap column (Acclaum PepMapTM 100, 75mm x 2cm), separated by the analytical column (EASY-Spray column, 75mm x 50cm, Thermo Fisher Scientific) with a gradient from 4 to 24% solvent B for 140min, 24 to 36% solvent B for 10 min at a flow rate 0.3 mL/min. The Orbitrap Eclipse Tribrid mass analyzer was operated in a top 10 data-dependent method. Full MS scans were acquired over the range m/z 400-1400 with mass resolution of 120,000 (at m/z 200). The AGC target value was 4.00E+05. The ten most intense peaks with charge state ≥2 was fragmented in the higher-energy collisional dissociation (HCD) collision cell with normalized collision energy of 35 and tandem mass spectra were acquired in the Orbitrap mass analyzer with a mass resolution of 30,000 at m/z 200.

**Protein identification and quantitation**

Database searching of all raw data files was performed in Proteome Discoverer 2.5 software (Thermo Fisher Scientific). SEQUEST-HT were used for database searching against Swissprot-Human database. Database searching against the corresponding reversed database was also performed to evaluate the false discovery rate (FDR) of peptide identification. The database searching parameters included precursor ion mass tolerance 10 ppm, fragment ion mass tolerance 0.02 Da, static modifications for carbamidomethyl cysteine (+57.021 Da / C) and TMTpro tags (+304.207 Da / K and N-terminal) and variable modifications for methionine oxidation (+15.995 Da / M). We obtained FDR of less than 1% on the peptide level and filtered with the high peptide confidence.

**Supplementary Table 1. Antibodies list.**

| **Purpose** | **Protein** | **Antibody Provider** | **Identifier**  **Cat No** | **Titer** | **Reactivity** | **Host** | **RRID** |
| --- | --- | --- | --- | --- | --- | --- | --- |
| Western  Blot | ULK1 | Cell signaling technology | 8054 | 1:1000 | human  mouse | rabbit | AB_11178668 |
|  | ULK2 | Thermo fisher scientific | PA5-22173 | 1:1000 | human  mouse | rabbit | AB_11153516 |
|  | ATG14 | Cell signaling technology |  | 1:1000 | mouse | rabbit |  |
|  | ATG14 | Cell signaling technology | 5504 | 1:1000 | human | rabbit | AB_10695397 |
|  | ATG14 | Genetex | GTX119950 | 1:1000 | human | rabbit | AB_10618570 |
|  | pATG14 | Cell signaling technology | 92340 | 1:1000 | human  mouse | rabbit | AB_2800182 |
|  | Cleaved capase3 | Cell signaling technology | 9661 | 1:1000 | human  mouse | rabbit | AB_2341188 |
|  | LC3B | Cell signaling technology | 2775 | 1:1000 | human  mouse | rabbit | AB_915950 |
|  | P62 | BD bioscience | 610832 | 1:1000 | human | mouse | AB_398151 |
|  | β-actin | Bethyl laboratories | A300-491A | 1:5000 | human  mouse | rabbit |  |
|  | GFP (mouse) | Santa cruz biotechnology | SC-9996 | 1:1000 | human  mouse | mouse | AB_627695 |
|  | GFP (rabbit) | Santa cruz biotechnology | SC-8334 | 1:1000 | human  mouse | rabbit | AB_641123 |
|  | Secondary antibody (rabbit) | Bethyl laboratories | A120-101P | 1:20000 | rabbit | goat |  |
|  | Secondary antibody (mouse) | Bethyl laboratories | A90-116P | 1:20000 | mouse | goat |  |
| FACS | CD16/CD32 | BD bioscience | BD553142 | 1:200 | - | - | AB_394656 |
|  | Fixable Viability Stain-Alexa 700 | BD bioscience | BD564997 | 1:10000 | - | - | AB_2869637 |
|  | CD45-BV785 | BD bioscience | BD564225 | 1:2000 | mouse | - | AB_2716861 |
|  | CD44-BV395 | BD bioscience | BD568507 | 1:200 | mouse | - |  |
|  | CD11b-BV510 | Biolegend | 101263 | 1:200 | mouse | - | AB_2629529 |
|  | CD11c-BV421 | Biolegend | 117343 | 1:200 | mouse | - | AB_2563099 |
|  | CD3-FITC | Biolegend | 100203 | 1:200 | mouse | - | AB_312660 |
|  | CD19-APC | Biolegend | 152409 | 1:200 | mouse | - | AB_2629838 |
|  | CD335-PE/Cy7 | Biolegend | 137617 | 1:200 | mouse | - | AB_11218594 |
|  | CD8a-PerCP/Cy5.5 | Biolegend | 100733 | 1:200 | mouse | - | AB_2075239 |
|  | CD4-PE | Biolegend | 100407 | 1:200 | mouse | - | AB_312692 |
|  | CD62L-APC/Cy7 | Biolegend | 104427 | 1:200 | mouse | - | AB_830798 |
|  | F4/80-PerCP/Cy5.5 | Biolegend | 123127 | 1:200 | mouse | - | AB_893496 |
|  | Ly6G-FITC | Biolegend | 127605 | 1:200 | mouse | - | AB_1236488 |
|  | Ly6C-PE | Biolegend | 128007 | 1:2000 | mouse | **-** | AB_1186133 |
|  | CD206-BV605 | Biolegend | 141721 | 1:200 | mouse | **-** | AB_2562340 |
|  | MHCII-BV496 | Invitrogen | 364-5321-80 | 1:200 | mouse | **-** |  |

| **Purpose** | **Protein** | **Antibody Provider** | **Identifier**  **Cat No** | **Titer** | **Reactivity** | **Host** | **RRID** |
| --- | --- | --- | --- | --- | --- | --- | --- |
| IHC | ULK1 | Genetex | GTX16974 | 1:400 | mouse  human | rabbit |  |
|  | pATG14 | Invitrogen | PA5-104573 | 1:400 | mouse  human | rabbit |  |
|  | LC3B | Abcam | ab51520 | 1:400 | mouse | rabbit | AB_881429 |
|  | P62 | Abcam | ab91526 | 1:200 | mouse | rabbit | AB_2050336 |
|  | CK19 | Abcam | ab133496 | 1:2500 | mouse | rabbit | AB_11155282 |
|  | Ki67 | Abcam | ab15580 | 1:750 | mouse | rabbit | AB_443209 |
|  | aSMA | Abcam | ab124964 | 1:2000 | mouse | rabbit | AB_11129103 |
|  | PDPN | Abcam | ab109059 | 1:2-500 | mouse  human | rabbit | AB_2848181 |
|  | FAP | Invitrogen | PA5-99458 | 1:100 | mouse  human | rabbit |  |
|  | CD3 | Abcam | ab16669 | 1:100 | mouse | rabbit | AB_443425 |
|  | CD4 | Abcam | ab183685 | 1:100 | mouse | rabbit | AB_2686917 |
|  | CD8a | Abcam | ab209775 | 1:100 | mouse | rabbit | AB_2860566 |
|  | CD8 | Akoya | OP000001 | 1:150 | human | rabbit |  |
|  | NCR1 | Abcam | ab214468 | 1:200 | mouse | rabbit | AB_2814876 |
|  | CD86 | Cell signaling technology | 19589 | 1:200 | mouse | rabbit | AB_2892094 |
|  | iNOS | Cell signaling technology | 68186 | 1:1000 | mouse | rabbit | AB_3662912 |
|  | CD204 | Thermo fisher scientific | MA5-29733 | 1:100 | mouse | rabbit | AB_2785556 |
|  | Ly6G | Abcam | ab238132 | 1:150 | mouse | rabbit | AB_2923218 |
|  | OPN/SPP1 | Abcam | ab218237 | 1:500 | mouse | rabbit | AB_2732079 |
|  | VEGFA | Genetex | GTX102643 | 1:500 | mouse  human | rabbit | AB_11174248 |

**Supplementary Table 2. Oligonucleotide sequences.**

| **Gene** | | **5` - Oligo Sequnces - 3`** | **Species** | **Purpose** |
| --- | --- | --- | --- | --- |
| *ULK1* | sense | CGGGCCCTGGATACGTCTTGTAATCTCGAGATTACAAGACGTATCCAGGGCTTTTTG | human | Knockdown |
|  | antisense | ATTCAAAAAGCCCTGGATACGTCTTGTAATCTCGAGATTACAAGACGTATCCAGGGC |  |  |
| *Ulk1* | sense | CACCGTTGTCTACCAGTGTCTGACA | mouse | Knockout |
|  | antisense | AAACTGTCAGACACTGGTAGACAAC |  |  |
| *Ulk1*-flox | forward | CTCCATGTGAGTTGGGCGGTAAAGG |  | genotyping |
|  | reverse | AAACATTCCCTCTGATGCCTACC |  |  |
| *Ccl2* | forward | CCCAATGAGTAGGCTGGAGA |  | qPCR |
|  | reverse | TCTGGACCCATTCCTTCTTG |  |  |
| *Cxcl1* | forward | CAAGGCTGGTCCATGCTCC |  |  |
|  | reverse | TGCTATCACTTCCTTTCTGTTGC |  |  |
| *Cxcl2* | forward | CCAACCACCAGGCTACAGG |  |  |
|  | reverse | GCGTCACACTCAAGCTCTG |  |  |
| *Cxcl12* | forward | TGCATCAGTGACGGTAAACCA |  |  |
|  | reverse | TTCTTCAGCCGTGCAACAATC |  |  |
| *Csf3* (G-CSF) | forward | ATCCCGAAGGCTTCCCTGAGTG |  |  |
|  | reverse | AGGAGACCTTGGTAGAGGCAGA |  |  |
| *ActB* | forward | ATCATTGCTCCTCCTGAGCG |  |  |
|  | reverse | CGGACTCATCGTACTCCTGC |  |  |

**Supplementary Table 3. KEGG pathways enrichment analysis of** **upregulated gene sets in *Ulk1* KO cells compared with *Ulk1* WT control cells.**

| **MapID** | **MapName** | **Number of SigGenes** | **Sig NotIn KEGG** | **Genome In KEGG** | **Genome NotIn KEGG** | **P Value** | **Bonferroni** | **FDR** |
| --- | --- | --- | --- | --- | --- | --- | --- | --- |
| 04010 | MAPK signaling pathway | 19 | 285 | 301 | 25688 | 1.889 E-08 | 5.553  E-06 | 5.553 E-06 |
| 04151 | PI3K-Akt signaling pathway | 19 | 285 | 364 | 25625 | 3.276 E-07 | 9.631  E-05 | 4.816 E-05 |
| 04060 | Cytokine-cytokine receptor interaction | 16 | 288 | 294 | 25695 | 2.164 E-06 | 6.362  E-04 | 9.423 E-05 |
| 04020 | Calcium signaling pathway | 15 | 289 | 253 | 25736 | 1.841 E-06 | 5.413  E-04 | 9.423 E-05 |
| 04970 | Salivary secretion | 9 | 295 | 86 | 25903 | 7.511 E-06 | 2.208  E-03 | 2.453 E-04 |
| 05412 | Arrhythmogenic right ventricular cardiomyopathy | 8 | 296 | 84 | 25905 | 5.745 E-05 | 1.689  E-02 | 1.152 E-03 |
| 03320 | PPAR signaling pathway | 8 | 296 | 89 | 25900 | 8.318 E-05 | 2.445  E-02 | 1.438 E-03 |
| 04148 | Efferocytosis | 10 | 294 | 161 | 25828 | 1.201 E-04 | 3.530  E-02 | 1.961 E-03 |

| **MapID** | **MapName** | **Gene ID** |
| --- | --- | --- |
| 04010 | MAPK signaling pathway | 11601, 11839, 12286, 12288, 12531, 13638, 14173, 15507, 18053, 18654, 18751, 19099, 19259, 20112, 239556, 269881, 53608, 78405, 81904 |
| 04151 | PI3K-Akt signaling pathway | 11601, 11839, 12575, 12835, 13638, 14173, 14702, 14745, 16190, 16399, 16421, 18053, 18127, 18414, 18654, 192897, 320207, 78134, 78405 |
| 04060 | Cytokine-cytokine receptor interaction | 12161, 12778, 16174, 16190, 17082, 18053, 18414, 20296, 20306, 20307, 20309, 21941, 225392, 50931, 53603, 57349 |
| 04020 | Calcium signaling pathway | 11548, 12062, 12286, 12288, 12494, 14173, 15235, 15465, 15565, 18125, 18127, 18751, 18795, 228550, 239556 |
| 04970 | Salivary secretion | 11548, 11830, 12494, 18125, 18751, 18795, 234889, 71395, 74180 |
| 05412 | Arrhythmogenic right ventricular cardiomyopathy | 12288, 13405, 16399, 16421, 16651, 18125, 192897, 81904 |
| 03320 | PPAR signaling pathway | 11807, 12895, 15360, 20249, 22259, 26457, 26569, 78070 |
| 04148 | Efferocytosis | 11846, 12895, 15170, 171284, 17289, 22174, 22259, 50770, 70417, 78070 |

**Supplementary Table 4. KEGG pathway enrichment analysis of upregulated gene sets in proteomics analysis from CQ- versus vehicle-treated HPNE KRAS^G12D^ cells.**

| **MapID** | **MapName** | **Number** | **List Total** | **Pop Hits** | **Pop Total** | **P Value** | **Benjamini** | **FDR** |
| --- | --- | --- | --- | --- | --- | --- | --- | --- |
| 04060 | Cytokine-cytokine receptor interaction | 23 | 164 | 298 | 8840 | 2.416  E-08 | 5.485  E-06 | 5.147  E-06 |
| 04350 | TGF-beta signaling pathway | 11 | 164 | 108 | 8840 | 2.824  E-05 | 3.205  E-03 | 3.008  E-03 |
| 04137 | Mitophagy - animal | 10 | 164 | 105 | 8840 | 1.307  E-04 | 9.892  E-03 | 9.282  E-03 |
| 04630 | JAK-STAT signaling pathway | 12 | 164 | 168 | 8840 | 2.661  E-04 | 1.510  E-02 | 1.417  E-02 |
| 05171 | Coronavirus disease - COVID-19 | 14 | 164 | 238 | 8840 | 4.424  E-04 | 2.009  E-02 | 1.885  E-02 |
| 04140 | Autophagy - animal | 11 | 164 | 169 | 8840 | 1.114  E-03 | 3.286  E-02 | 3.083  E-02 |
| 04610 | Complement and coagulation cascades | 8 | 164 | 88 | 8840 | 1.156  E-03 | 3.286  E-02 | 3.083  E-02 |

| **MapID** | **MapName** | **Genes** |
| --- | --- | --- |
| 04060 | Cytokine-cytokine receptor interaction | IL4R, BMPR2, CXCL8, TNFRSF12A, IFNGR1, IL10RB, TNFRSF19, TNFRSF10B, INHBA, ACVR1B, OSMR, TGFBR1, ACVR2A, TGFBR2, TNFRSF10D, TNFRSF1A, LTBR, IL6ST, IL7R, RELT, TNFRSF21, IL13RA1, BMPR1A |
| 04350 | TGF-beta signaling pathway | BMPR2, FST, INHBA, ACVR1B, THBS1, LTBP1, TGFBR1, ACVR2A, BMPR1A, FBN1, TGFBR2 |
| 04137 | Mitophagy - animal | GABARAPL2, MAP1LC3B, MAP1LC3A, UBB, CALCOCO2, BNIP3, NBR1, TAX1BP1, SQSTM1, GABARAP |
| 04630 | JAK-STAT signaling pathway | PDGFRB, PDGFRA, IL4R, IFNGR1, IL10RB, PDGFB, IL6ST, IL7R, OSMR, SOS2, JAK1, IL13RA1 |
| 05171 | Coronavirus disease - COVID-19 | CXCL8, TNFRSF1A, C4B, RPL36AL, RPS29, RPL14, AGTR1, RPL38, FAU, RPL29, IL6ST, RPS24, JAK1, HBEGF |
| 04140 | Autophagy - animal | GABARAPL2, MAP1LC3B, MAP1LC3A, UBB, CALCOCO2, BNIP3, NBR1, TAX1BP1, SQSTM1, PRKACB, GABARAP |
| 04610 | Complement and coagulation cascades | C4B, SERPINE2, PLAU, BDKRB1, PLAT, A2M, CD46, CLU |

**Supplementary Table 5. DAVID Functional Annotation Analysis from gene sets negatively expressed with ULK1 from PAAD in TCGA (**QCMG; *Nature 2016***).**

| **Term** | **Count** | **List Total** | **Pop Hits** | **Pop Total** | **PValue** | **Benjamini** | **FDR** |
| --- | --- | --- | --- | --- | --- | --- | --- |
| GO:0019886~antigen processing and presentation of exogenous peptide antigen via MHC class II | 17 | 1106 | 31 | 19734 | 1.14  E-12 | 2.47  E-09 | 2.45  E-09 |
| GO:0002503~peptide antigen assembly with MHC class II protein complex | 13 | 1106 | 16 | 19734 | 1.32  E-12 | 2.47  E-09 | 2.45  E-09 |
| GO:0042776~proton motive force-driven mitochondrial ATP synthesis | 23 | 1106 | 67 | 19734 | 5.66  E-12 | 7.04  E-09 | 6.97  E-09 |
| GO:0002504~antigen processing and presentation of peptide or polysaccharide antigen via MHC class II | 13 | 1106 | 20 | 19734 | 7.43  E-11 | 6.94  E-08 | 6.87  E-08 |
| GO:0019882~antigen processing and presentation | 17 | 1106 | 45 | 19734 | 1.16  E-09 | 8.68  E-07 | 8.59  E-07 |
| GO:0045333~cellular respiration | 16 | 1106 | 40 | 19734 | 1.63  E-09 | 1.01  E-06 | 1.00  E-06 |
| GO:0006955~immune response | 64 | 1106 | 505 | 19734 | 1.97  E-09 | 1.05  E-06 | 1.04  E-06 |
| GO:0009060~aerobic respiration | 20 | 1106 | 68 | 19734 | 3.43  E-09 | 1.60  E-06 | 1.58  E-06 |
| GO:0050870~positive regulation of T cell activation | 15 | 1106 | 38 | 19734 | 7.49  E-09 | 3.11  E-06 | 3.07  E-06 |
| GO:0050778~positive regulation of immune response | 14 | 1106 | 37 | 19734 | 5.01  E-08 | 1.73  E-05 | 1.71  E-05 |
| GO:1902600~proton transmembrane transport | 27 | 1106 | 139 | 19734 | 5.10  E-08 | 1.73  E-05 | 1.71  E-05 |
| GO:0032543~mitochondrial translation | 21 | 1106 | 98 | 19734 | 4.07  E-07 | 1.27  E-04 | 1.25  E-04 |
| GO:0006120~mitochondrial electron transport, NADH to ubiquinone | 14 | 1106 | 46 | 19734 | 8.93  E-07 | 2.57  E-04 | 2.54  E-04 |
| GO:0002250~adaptive immune response | 51 | 1106 | 452 | 19734 | 3.42  E-06 | 9.02  E-04 | 8.92  E-04 |
| GO:0032760~positive regulation of tumor necrosis factor production | 21 | 1106 | 112 | 19734 | 3.68  E-06 | 9.02  E-04 | 8.92  E-04 |
| GO:0045040~protein insertion into mitochondrial outer membrane | 8 | 1106 | 14 | 19734 | 4.11  E-06 | 9.02  E-04 | 8.92  E-04 |
| GO:0006122~mitochondrial electron transport, ubiquinol to cytochrome c | 8 | 1106 | 14 | 19734 | 4.11  E-06 | 9.02  E-04 | 8.92  E-04 |
| GO:0032981~mitochondrial respiratory chain complex I assembly | 15 | 1106 | 67 | 19734 | 1.65  E-05 | 3.42  E-03 | 3.39  E-03 |
| GO:0006119~oxidative phosphorylation | 7 | 1106 | 12 | 19734 | 2.10  E-05 | 4.12  E-03 | 4.08  E-03 |
| GO:0032729~positive regulation of type II interferon production | 16 | 1106 | 78 | 19734 | 2.38  E-05 | 4.44  E-03 | 4.40  E-03 |
| GO:0042102~positive regulation of T cell proliferation | 14 | 1106 | 61 | 19734 | 2.63  E-05 | 4.68  E-03 | 4.64  E-03 |
| GO:0071346~cellular response to type II interferon | 18 | 1106 | 103 | 19734 | 5.53  E-05 | 9.38  E-03 | 9.28  E-03 |
| GO:0006123~mitochondrial electron transport, cytochrome c to oxygen | 9 | 1106 | 26 | 19734 | 5.95  E-05 | 9.66  E-03 | 9.56  E-03 |

**DAVID Gene Ontology analysis of differentially expressed genes showing top 20 upregulated biological process in which negatively expressed in *Ulk1* from PAAD in TCGA based on *P* value.**

| **Term** | **Genes** |
| --- | --- |
| GO:0019886~antigen processing and presentation of exogenous peptide antigen via MHC class II | CD74, HLA-DRB5, FCER1G, IFI30, CTSS, HLA-DMA, HLA-DMB, HLA-DPB1, HLA-DRA, HLA-DOA, HLA-DOB, B2M, HLA-DQA1, HLA-DQB2, HLA-DRB1, HLA-DPA1, HLA-DQB1 |
| GO:0002503~peptide antigen assembly with MHC class II protein complex | HLA-DRB5, HLA-DMA, HLA-DMB, HLA-DPB1, HLA-DRA, HLA-DOA, HLA-DOB, B2M, HLA-DQA1, HLA-DQB2, HLA-DRB1, HLA-DPA1, HLA-DQB1 |
| GO:0042776~proton motive force-driven mitochondrial ATP synthesis | ATP5PF, NDUFA13, NDUFA7, ATP5PB, NDUFB5, NDUFA3, NDUFB3, NDUFA2, NDUFA1, SDHC, SDHD, SDHA, ATP5F1C, SDHB, ATP5F1A, NDUFS6, NDUFS5, NDUFAB1, NDUFS3, ATP5PO, NDUFS2, NDUFS1, ATP5MG |
| GO:0002504~antigen processing and presentation of peptide or polysaccharide antigen via MHC class II | HLA-DRB5, HLA-B, HLA-DMA, HLA-DMB, HLA-DPB1, HLA-DRA, HLA-DOA, HLA-DOB, HLA-DQA1, HLA-DQB2, HLA-DRB1, HLA-DPA1, HLA-DQB1 |
| GO:0019882~antigen processing and presentation | CD74, HLA-DRB5, HLA-B, HLA-C, PSMB8, CTSS, HLA-DMA, HLA-DMB, CD8A, HLA-DPB1, HLA-DRA, HLA-DOB, HLA-DQA1, HLA-DRB1, HLA-DPA1, HLA-DQB1, RAB8B |
| GO:0045333~cellular respiration | COX8A, COX7B, UQCRB, NDUFA4, COX4I1, COX7A2, UQCR10, COX5B, COX5A, COX7C, UQCRQ, UQCRC1, CYCS, NDUFS1, UQCRC2, SLC25A13 |
| GO:0006955~immune response | FCGR1BP, CD86, CXCL9, GPR65, LST1, SECTM1, FASLG, CTSS, TNFSF13B, IGLV2-8, C1QBP, IGKV2-24, CCR5, HLA-DOB, B2M, IGHV3-9, CTSC, CCR2, HLA-DPA1, IGHV3-30, HLA-B, HLA-C, PDCD1LG2, TNFRSF1B, HLA-E, CD8A, LCP2, TLR4, HLA-DQB2, HLA-DQB1, TLR2, CX3CR1, IGSF6, MARCHF1, PTAFR, CXCR4, CXCR6, SAMHD1, CD79B, HLA-DMA, IGLV1-40, HLA-DMB, IGLV6-57, IGLV5-45, IGLV3-21, CCL4, ICOS, GBP2, HLA-DQA1, MICB, CCR1, HLA-DRB5, IGHV3-11, IGKV1-5, TNFSF13, LILRB2, CD4, IL7, PRKRA, HLA-DPB1, HLA-DRA, TNFSF8, HLA-DRB1, C1QC |
| GO:0009060~aerobic respiration | NDUFA13, NDUFA7, UQCRB, NDUFB5, NDUFA3, NDUFB3, NDUFA2, NDUFA1, SDHC, SDHB, NDUFS6, NDUFS5, NDUFAB1, CAT, NDUFS3, UQCRC1, MFN2, NDUFS2, NDUFS1, UQCRC2 |
| GO:0050870~positive regulation of T cell activation | HLA-DRB5, HLA-DMA, CD4, HLA-DMB, HLA-DPB1, HLA-DRA, HLA-DOA, HLA-DOB, B2M, HLA-DQA1, HLA-DQB2, HLA-DRB1, CCR2, HLA-DPA1, HLA-DQB1 |
| GO:0050778~positive regulation of immune response | HLA-DRB5, RSAD2, HLA-DMA, HLA-DMB, HLA-DPB1, HLA-DRA, HLA-DOA, HLA-DOB, B2M, HLA-DQA1, HLA-DQB2, HLA-DRB1, HLA-DPA1, HLA-DQB1 |
| GO:1902600~proton transmembrane transport | COX7B, SLC46A1, COX4I1, ATP5MC3, UQCR10, COX5B, COX5A, ATP5MC1, ATP5F1A, UQCRFS1, ATP6V1E1, SLC15A3, ATP5MG, ATP6V1F, COX8A, ATP5PF, ATP6V0B, ATP6V0E1, SLC16A1, ATP5PB, ATP5F1C, SLC9B2, SLC9A6, ATP6V1B2, UQCRC1, ATP5PO, SLC25A13 |
| GO:0032543~mitochondrial translation | MRPS17, NDUFA7, MRPS27, RARS1, FASTKD2, MRPS14, MRPS12, MRPL19, MRPL16, MRPL39, MRPS21, MRPL36, MRPL37, MRPL34, MRPL24, MRPL35, MRPL11, MRPL20, MRPS9, IARS2, AURKAIP1 |
| GO:0006120~mitochondrial electron transport, NADH to ubiquinone | NDUFA7, NDUFB5, NDUFA4, NDUFA3, NDUFB3, NDUFA2, NDUFA1, COQ9, NDUFS6, NDUFS5, NDUFAB1, NDUFS3, NDUFS2, NDUFS1 |
| GO:0002250~adaptive immune response | CD86, CD84, CLEC10A, TRAC, CD3E, CD3D, CTSS, IGLV2-8, SIT1, C1QBP, IGKV2-24, HLA-DOA, HLA-DOB, HAVCR2, HLA-DPA1, SYK, BTN3A1, THEMIS, HLA-B, HLA-C, TAP1, PDCD1LG2, MPEG1, HLA-E, CLEC4A, CD8A, BTK, HLA-DQB2, HLA-DQB1, CX3CR1, CD79B, HLA-DMA, IGLV1-40, HLA-DMB, IGLV6-57, IGLV5-45, IGLV3-21, HLA-DQA1, MICB, SLAMF1, HLA-DRB5, TRAT1, IGKV1-5, LILRB2, MCOLN2, LILRB4, CD4, HLA-DPB1, HLA-DRA, CD247, HLA-DRB1 |
| GO:0032760~positive regulation of tumor necrosis factor production | SASH3, CD84, SYK, DHX9, PTAFR, CYBB, SELENOK, HLA-E, IFIH1, CD2, TYROBP, PTPRC, CLEC7A, BTK, TNFRSF8, CD14, TLR4, TLR3, CCR2, HAVCR2, TLR2 |
| GO:0045040~protein insertion into mitochondrial outer membrane | TOMM70, MTCH2, SAMM50, MTX2, HSPA4, TOMM7, TOMM20, TOMM22 |
| GO:0006122~mitochondrial electron transport, ubiquinol to cytochrome c | UQCRB, UQCRQ, UQCRC1, UQCRFS1, CYCS, UQCR10, UQCRC2, UQCRHL |
| GO:0032981~mitochondrial respiratory chain complex I assembly | NDUFA13, DMAC1, NDUFB5, NDUFA3, NDUFB3, NDUFA2, NDUFA1, NDUFS5, NDUFAF4, NDUFAB1, NDUFAF2, TMEM126A, NDUFS3, NDUFS2, NDUFS1 |
| GO:0006119~oxidative phosphorylation | MSH2, UQCRB, C2ORF69, UQCRC1, CHCHD10, UQCRC2, ATP5F1C |
| GO:0032729~positive regulation of type II interferon production | SASH3, BTN3A1, BTN3A2, CD3E, CD2, CLEC7A, HLA-DPB1, TLR8, SLAMF6, CD14, TLR4, TLR3, CCR2, HAVCR2, SLAMF1, HLA-DPA1 |
| GO:0042102~positive regulation of T cell proliferation | SASH3, CD86, LILRB2, PDCD1LG2, SELENOK, CD3E, AIF1, TNFSF13B, HLA-DMB, PTPRC, HLA-DPB1, NCKAP1L, HAVCR2, HLA-DPA1 |
| GO:0071346~cellular response to type II interferon | GBP5, STXBP3, STAT1, FASLG, AIF1, EPRS1, CCL4, CASP1, CALM3, CARD16, GBP2, GBP1, TLR4, GBP4, TLR3, HLA-DPA1, TLR2, VAMP3 |
| GO:0006123~mitochondrial electron transport, cytochrome c to oxygen | COX8A, COX7B, NDUFA4, COX4I1, CYCS, COX7A2, COX5B, COX5A, COX7C |

**Supplementary Table 6. Differentially expressed genes (DEGs) between Ulk1 KO cells and Ulk1 WT control cells under nutrient complete (Com) or amino acid starvation (-AA), showing log2 fold changes and p-values (significance threshold: (|log2FC| > 2 and p < 0.05).**

**
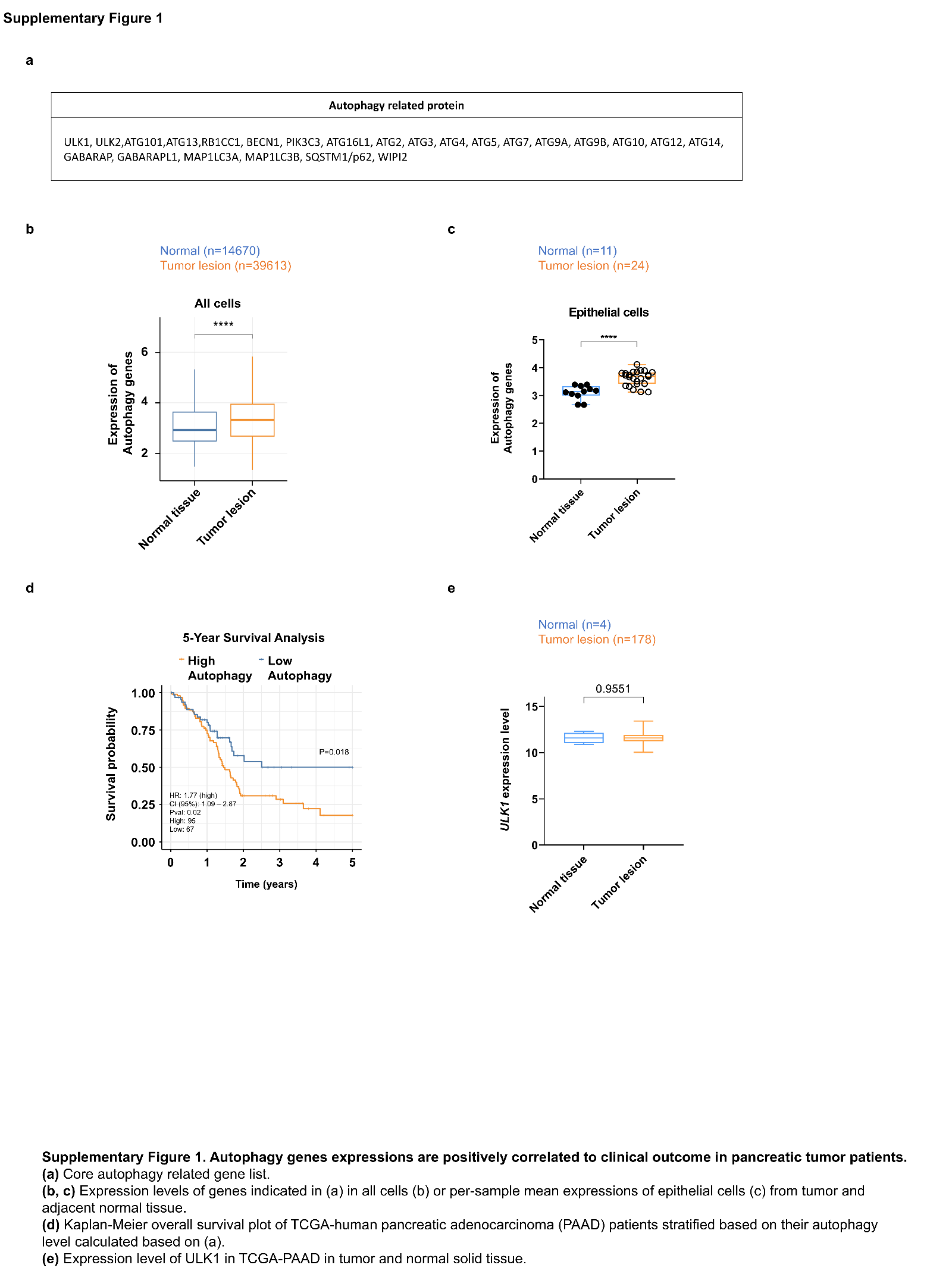
**


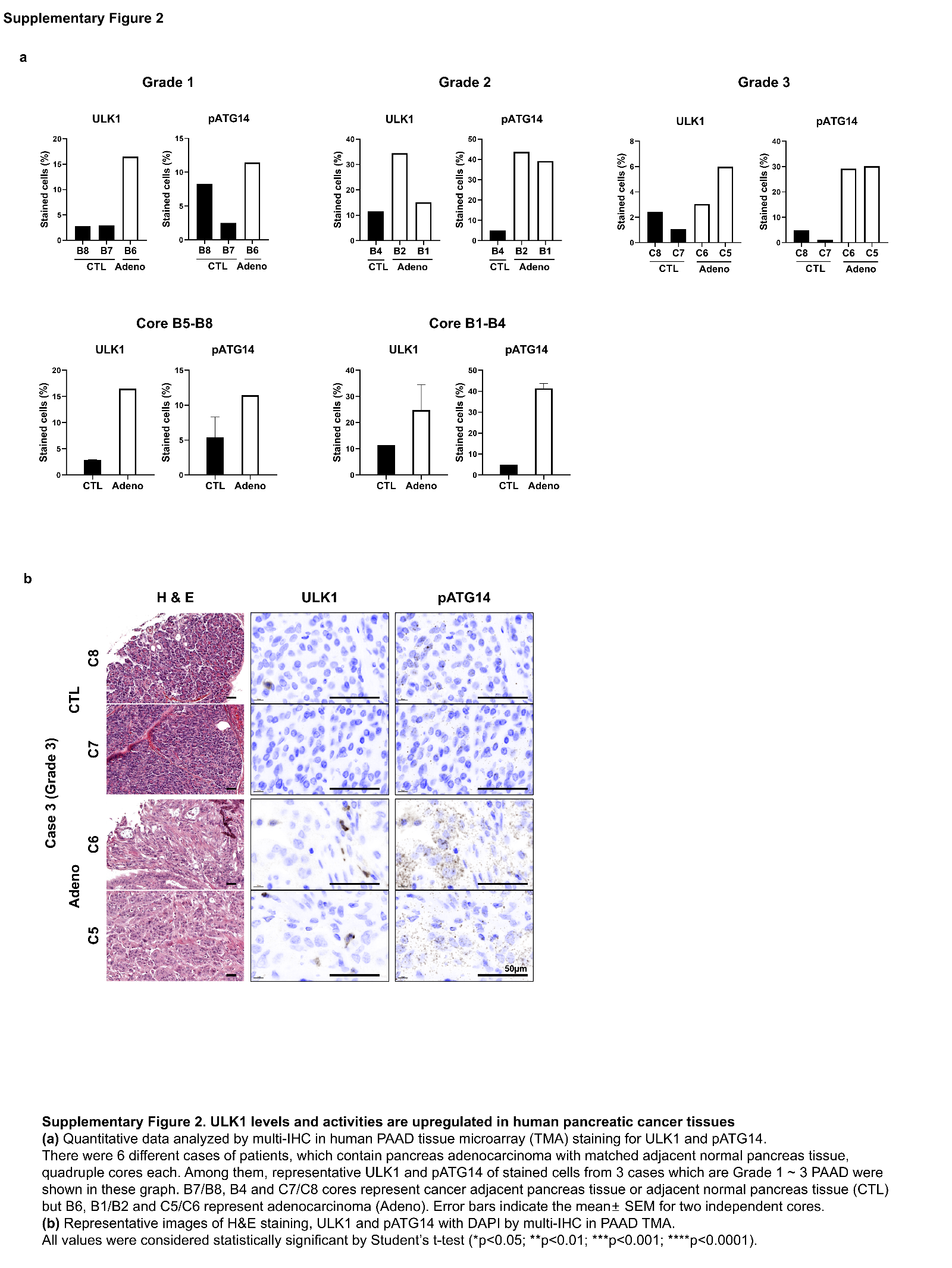


**
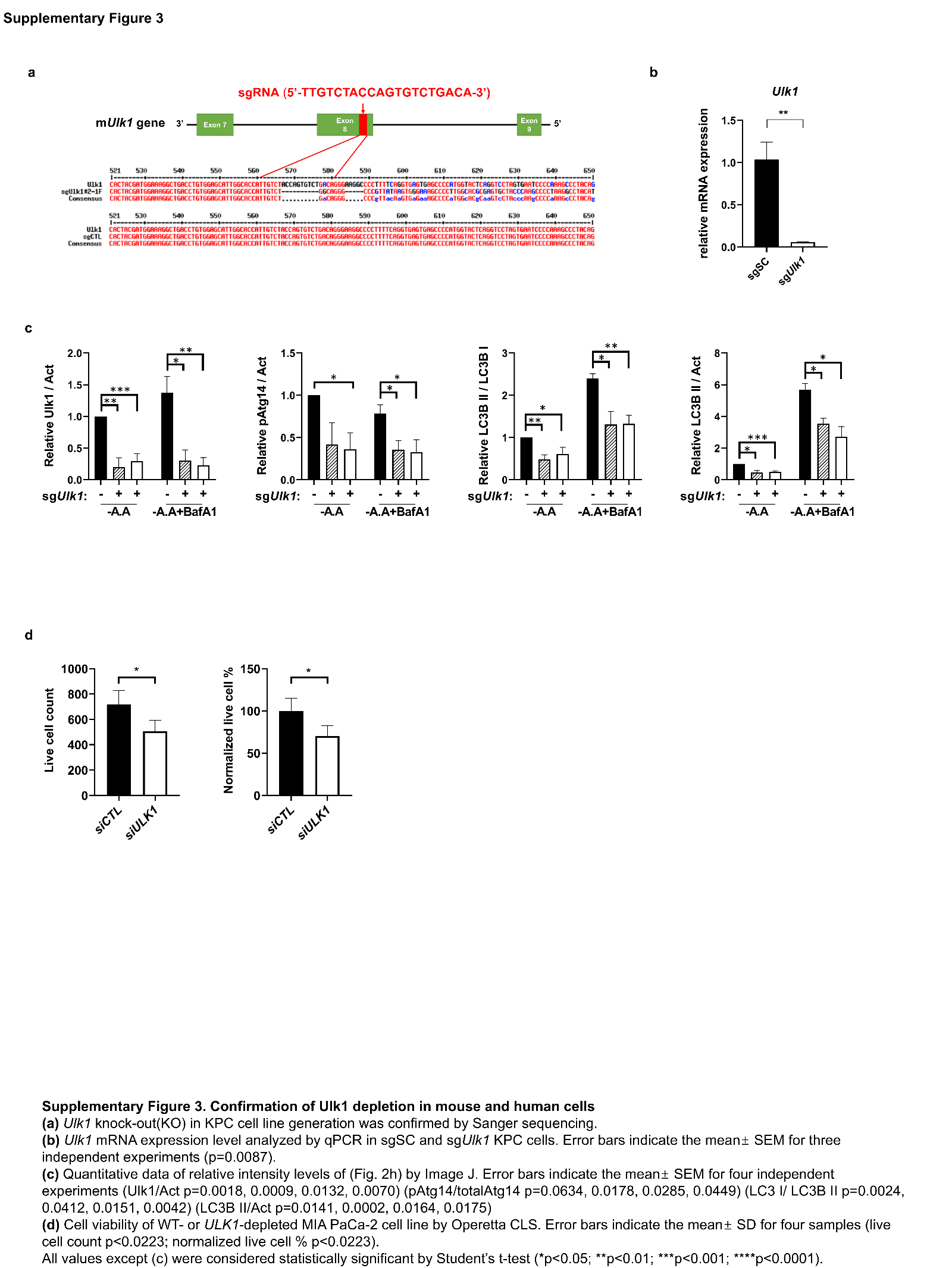
**

**
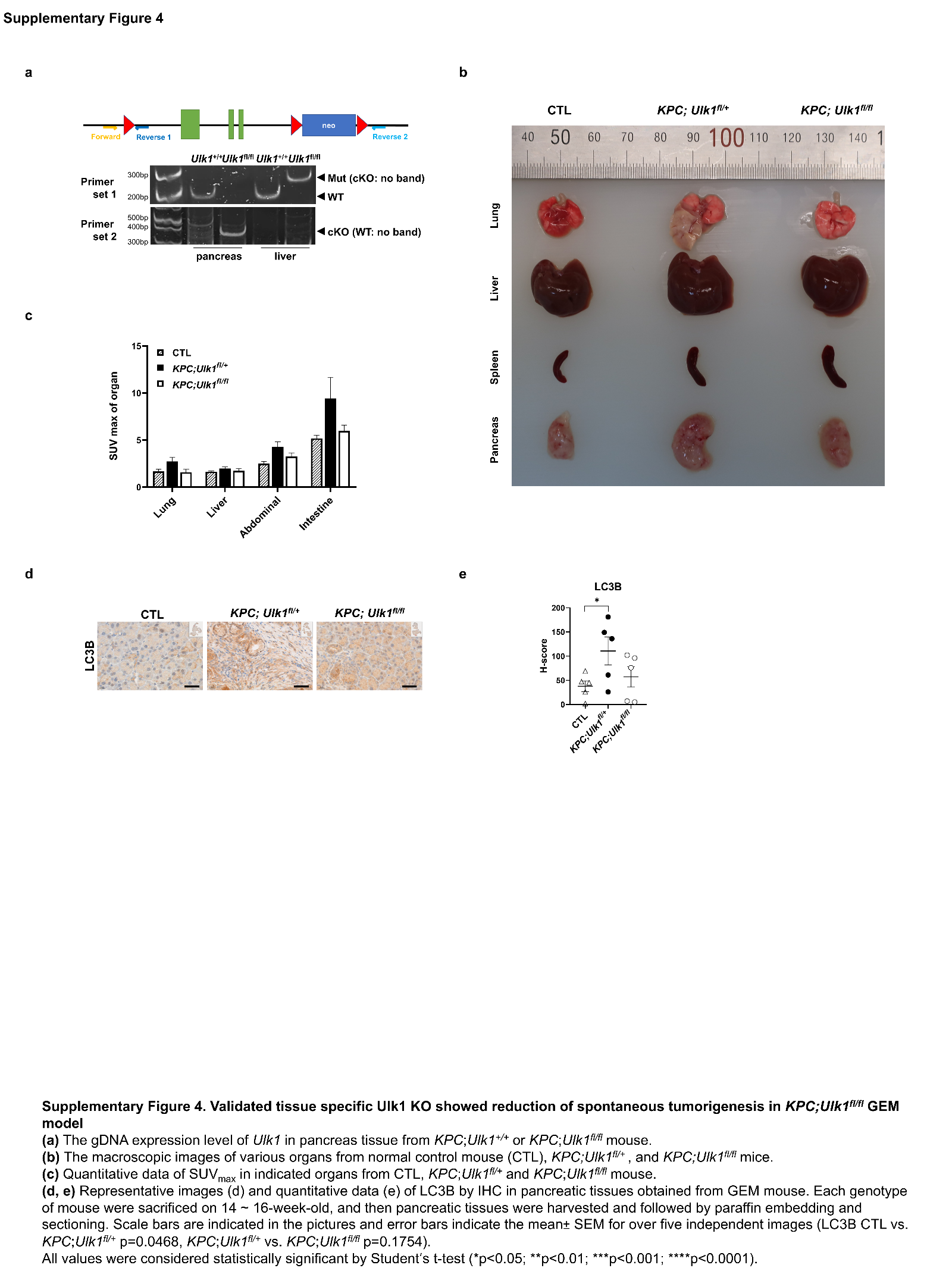
**

**
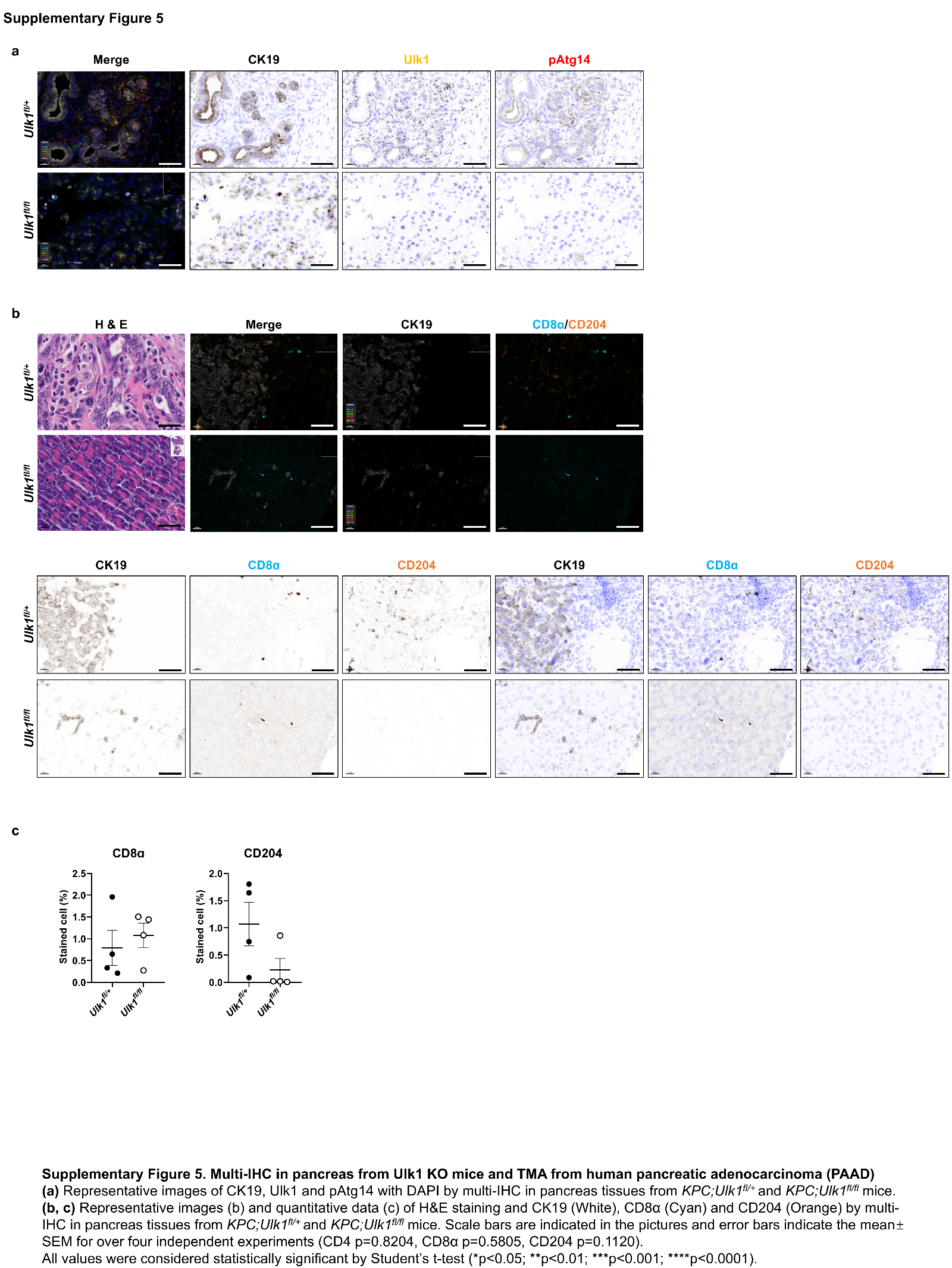
**

**
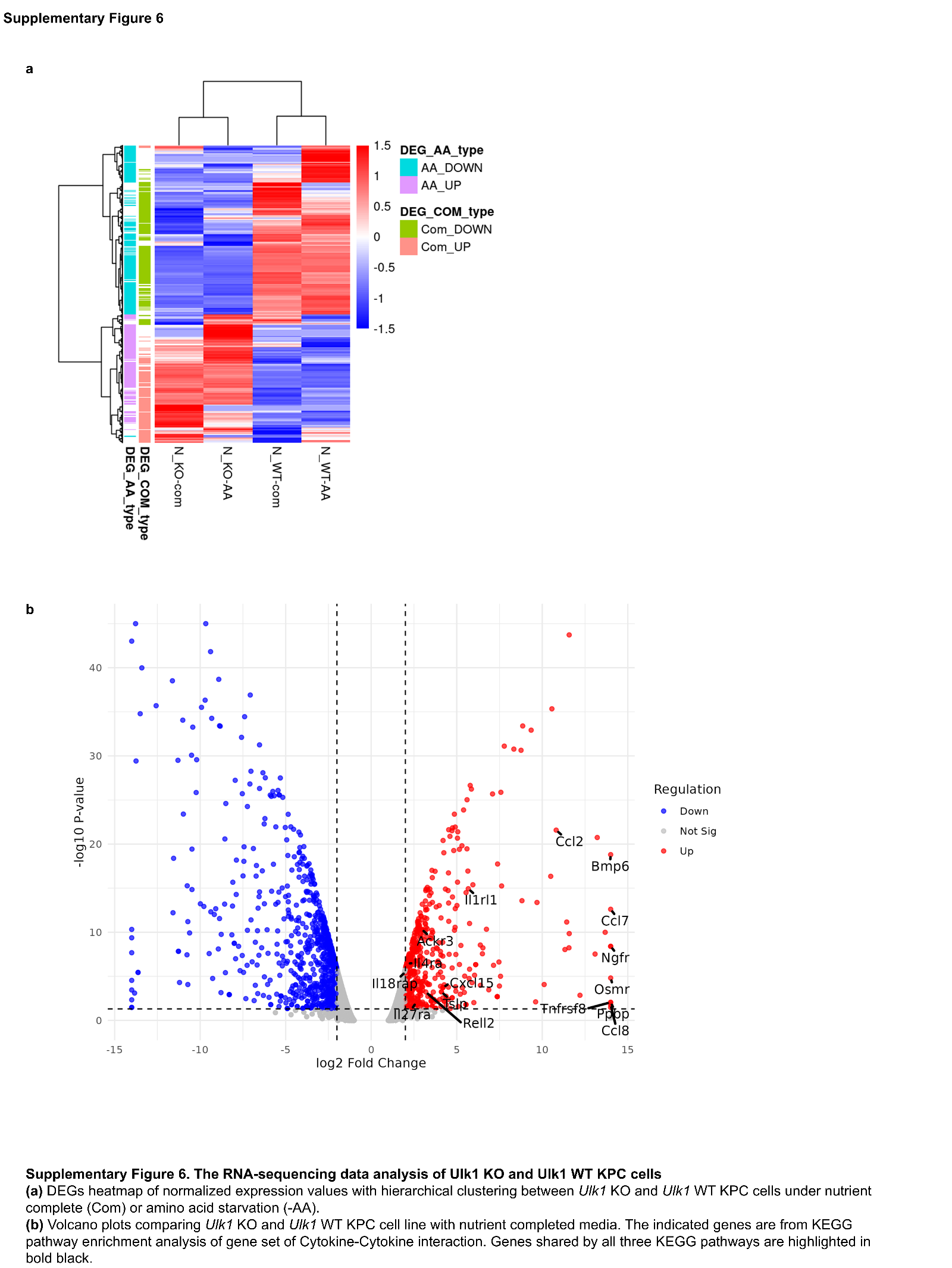
**

**
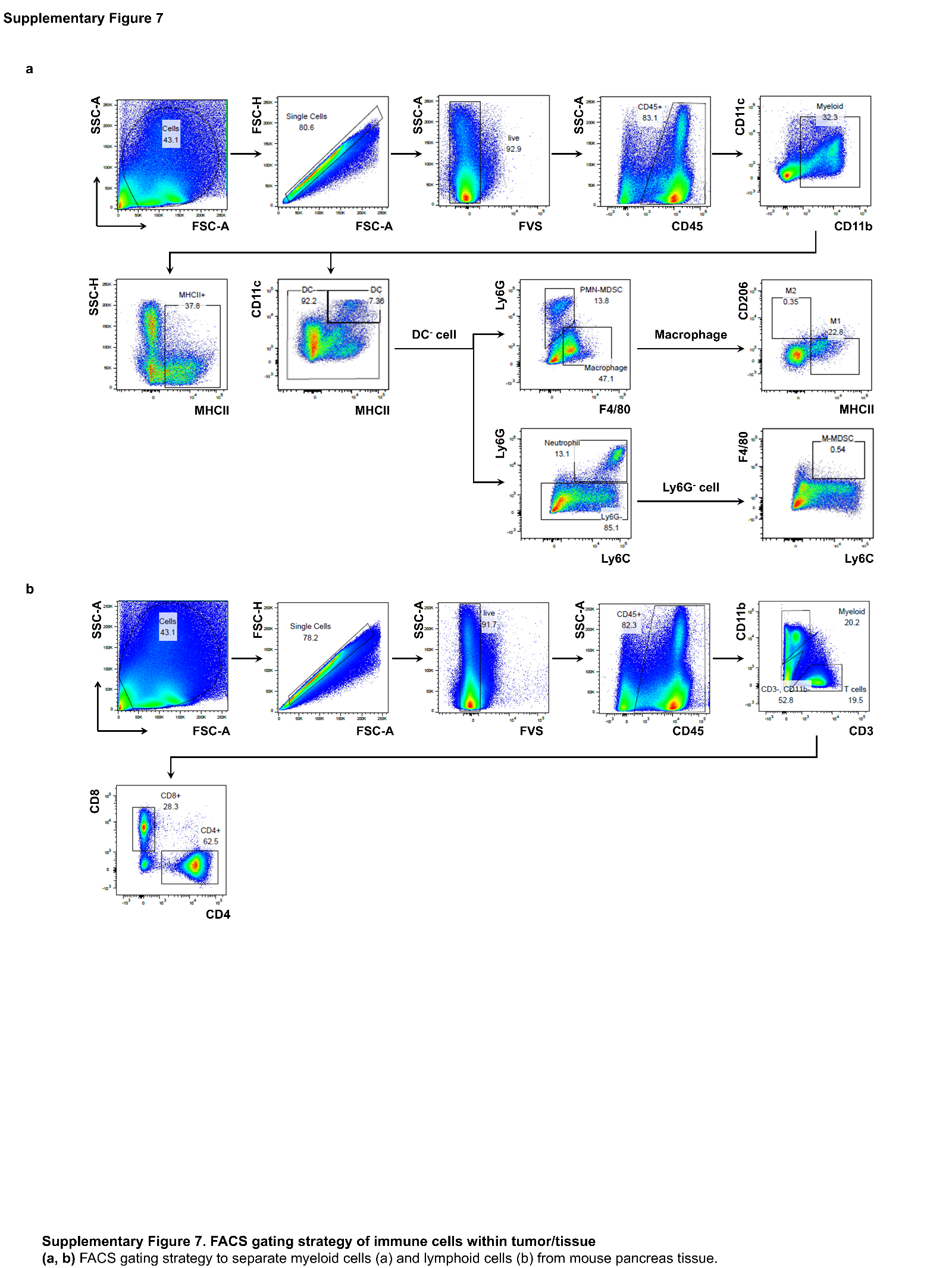
**

**
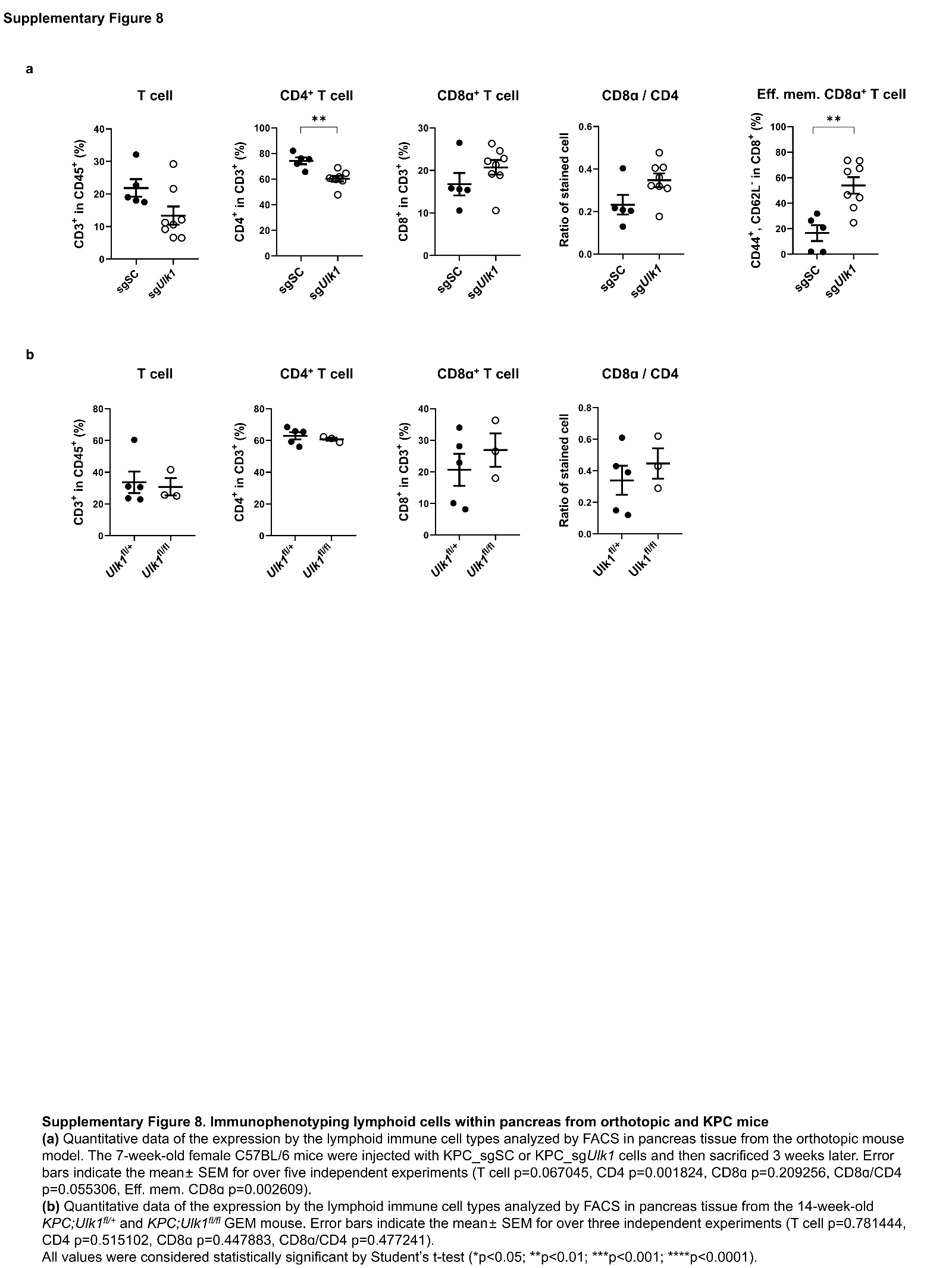
**

**
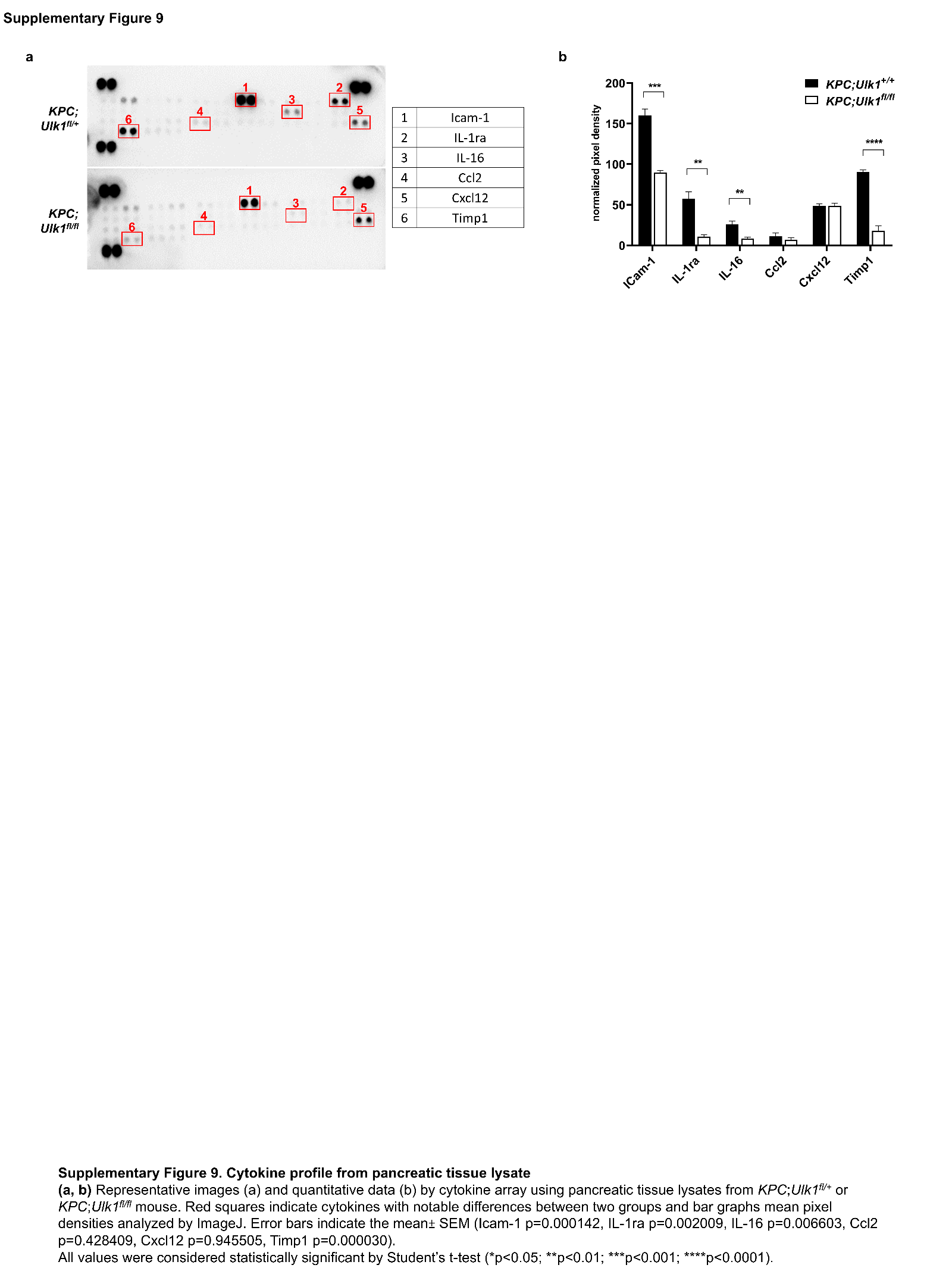
**

**
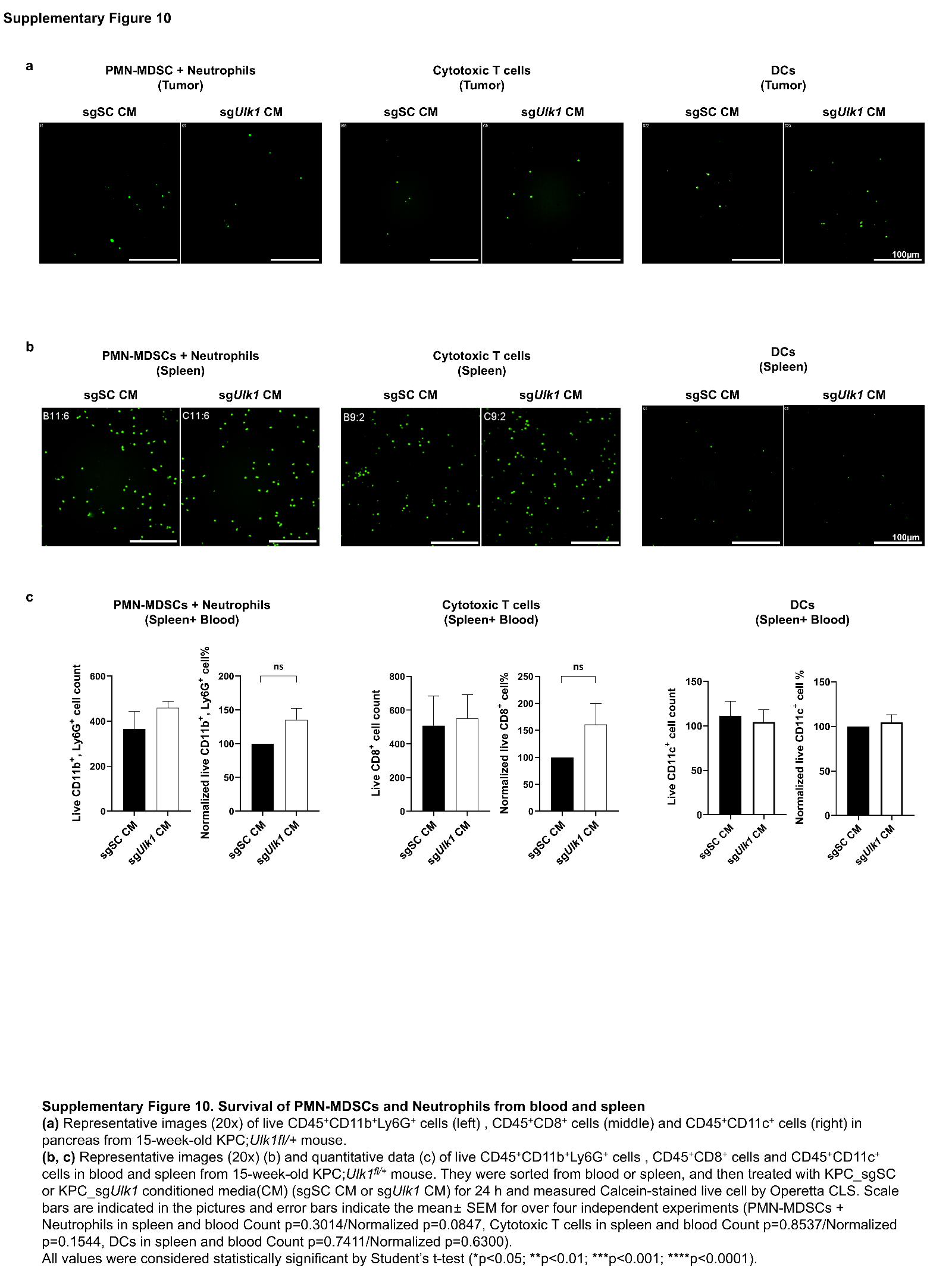
**

**
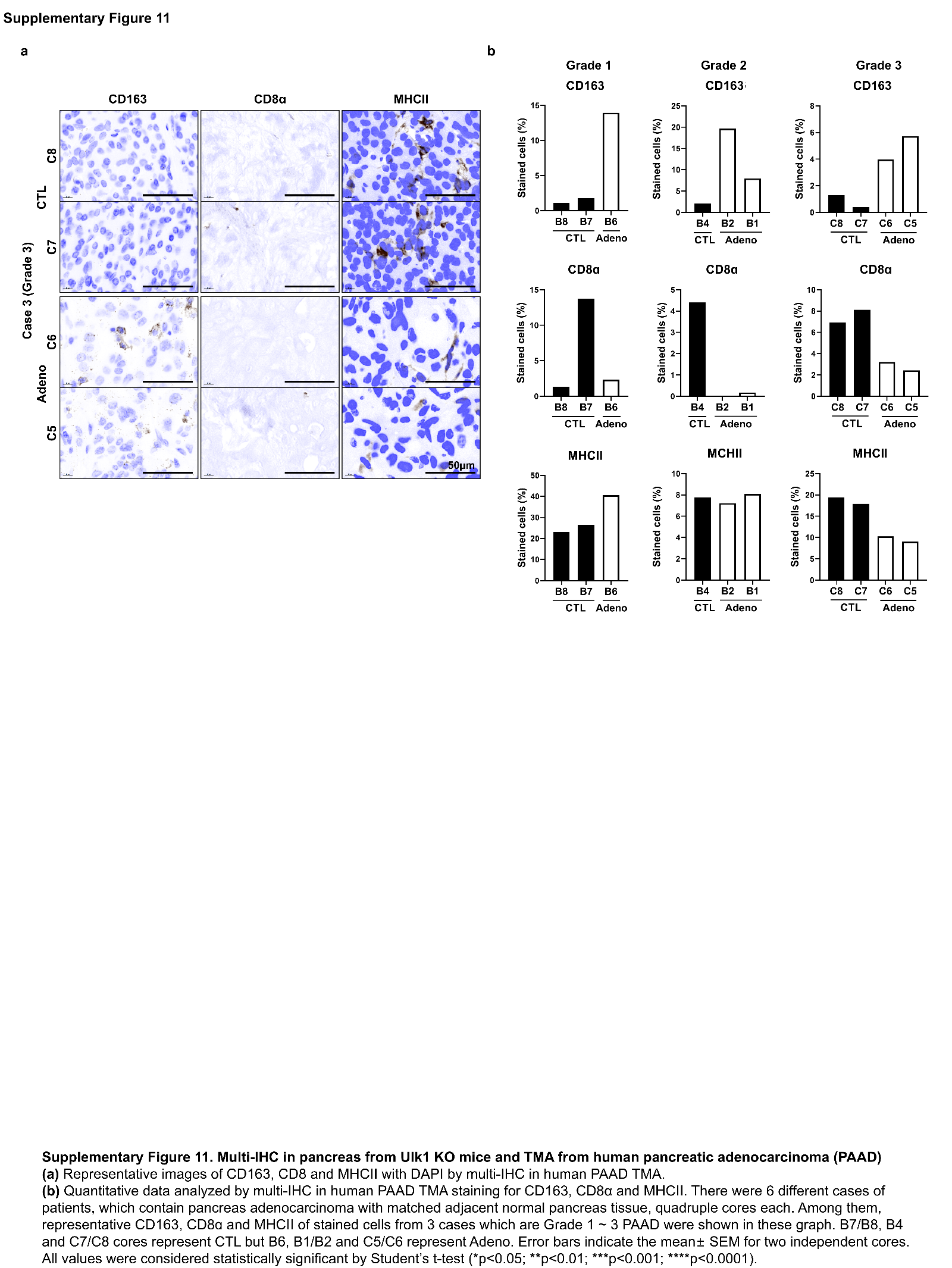
**
